# Smallest near-infrared fluorescent protein evolved from cyanobacteriochrome as versatile tag for spectral multiplexing

**DOI:** 10.1038/s41467-018-08050-8

**Published:** 2019-01-17

**Authors:** Olena S. Oliinyk, Anton A. Shemetov, Sergei Pletnev, Daria M. Shcherbakova, Vladislav V. Verkhusha

**Affiliations:** 1 Medicum, Faculty of Medicine, University of Helsinki, 00290 Helsinki, Finland; 2 Department of Anatomy and Structural Biology, and Gruss-Lipper Biophotonics Center, Albert Einstein College of Medicine, Bronx, NY 10461 USA; 3 Basic Science Program, Macromolecular Crystallography Laboratory, Frederick National Laboratory for Cancer Research sponsored by the National Cancer Institute, Frederick, MD 21702 USA

## Abstract

From a single domain of cyanobacteriochrome (CBCR) we developed a near-infrared (NIR) fluorescent protein (FP), termed miRFP670nano, with excitation at 645 nm and emission at 670 nm. This is the first CBCR-derived NIR FP evolved to efficiently bind endogenous biliverdin chromophore and brightly fluoresce in mammalian cells. miRFP670nano is a monomer with molecular weight of 17 kDa that is 2-fold smaller than bacterial phytochrome (BphP)-based NIR FPs and 1.6-fold smaller than GFP-like FPs. Crystal structure of the CBCR-based NIR FP with biliverdin reveals a molecular basis of its spectral and biochemical properties. Unlike BphP-derived NIR FPs, miRFP670nano is highly stable to denaturation and degradation and can be used as an internal protein tag. miRFP670nano is an effective FRET donor for red-shifted NIR FPs, enabling engineering NIR FRET biosensors spectrally compatible with GFP-like FPs and blue–green optogenetic tools. miRFP670nano unlocks a new source of diverse CBCR templates for NIR FPs.

## Abstract (web summary)

Near-infrared (NIR) fluorescent proteins (FPs) offer advantages for spectral multiplexing and deep-tissue imaging. Here the authors engineer a smaller NIR FP based on the unexplored cyanobacteriochrome photoreceptor and demonstrate its use in various applications in cell culture as well as whole-body imaging in vivo in mice.

## Introduction

Light absorption and fluorescence of green fluorescent protein (GFP)-like fluorescent proteins (FPs) are limited to a visible range of optical spectrum. Therefore, near-infrared (NIR) FPs and NIR biosensors are in high demand not only for deep-tissue in vivo imaging^1^ but, even more importantly, for spectral multiplexing with biosensors based on GFP-like FPs and common optogenetic tools based on opsins, LOV and CRY domains that are activatable with blue-green light^2^.

Bacterial photoreceptors have absorbance spectra in the NIR range due to covalently attached heme-derived linear tetrapyrrole compounds and allow engineering NIR FPs^1^. Several photoreceptors from a class of bacterial phytochrome photoreceptors (BphPs) were developed into bright monomeric NIR FPs, which efficiently bind endogenous biliverdin (BV) tetrapyrrole in mammalian cells^3–5^. However, the BphP-derived NIR FPs minimally require two domains, a PAS and a GAF, to covalently attach a BV chromophore and also possess a complex “figure-of-eight knot” structure topologically linking the GAF and PAS domains, which affects their folding^1^. Another class of bacterial photoreceptors, allophycocyanins (APCs), was also used to engineer NIR FPs, such as smURFP from TeAPC and several BDFPs from ApcF. Although the APC-based NIR FPs are smaller, they have low efficiency of BV binding, resulting in significantly lower brightness in mammalian cells than the BphP-derived NIR FPs^6–8^.

To overcome the drawbacks of the BphP- and APC-based NIR FPs, we turned our attention to a class of cyanobacteriochrome (CBCR) photoreceptors found in cyanobacteria^9^. Typical CBCRs consist of one or more GAF domains and effector domains^1,9^. GAF domains of CBCRs have several unique properties to consider them for engineering of NIR FPs. First, a single CBCR GAF domain is sufficient for autocatalytic binding of tetrapyrrole chromophore^10^, potentially allowing to engineer single-domain FPs, twice smaller than the  BphP-derived FPs. This binding occurs via a conserved Cys residue located in the GAF domain, in contrast to the Cys in the PAS domain in BphPs. Second, GAF domains of CBCRs are naturally monomeric^11,12^, unlike typically dimeric BphPs and oligomeric APCs^1^. Third, in contrast to BphPs and APCs, various CBCR subclasses exhibit a large spectral diversity and, moreover, a variety of photocycles in which GAF domains reversibly photoconvert between ultraviolet (UV)/blue-, blue/green-, green/red-, and red/NIR-absorbing forms^13,14^. Fourth, CBCR GAF domains are also found as components of complex signaling proteins^15^, suggesting that their structural fold is naturally optimized to use in fusion constructs^14^.

Despite these advantages, CBCRs utilize phycocyanobilin (PCB) tetrapyrrole as a chromophore. PCB is naturally present in plant and cyanobacteria but not in mammalian cells, which produce BV^3,16,17^. Recently, however, three CBCR GAF domains from *Acaryochloris marina* were shown to bind both PCB and BV^18–20^. Moreover, GAF domains in the  BphP-derived NIR FPs were adopted to covalently bind BV^21,22^. Based on these findings, we hypothesized that CBCRs can be engineered into BV-binding NIR FPs.

Here, we expressed various CBCRs in BV-producing *Escherichia coli* bacteria and found that the GAF domain of NpR3784 CBCR^23^ weakly binds BV and can be a template for NIR FP engineering. We next subject NpR3784 GAF to multiple rounds of molecular evolution, which resulted in the first CBCR-derived NIR FP. Importantly, similar to the  BphP-based FPs, the CBCR-derived NIR FP brightly fluoresces in mammalian cells without supplementation of exogenous BV chromophore. Characterization of the developed NIR FP showed its numerous advantages over NIR FPs developed from other photoreceptors, including monomeric state, substantially smaller size, significantly higher protein stability in vitro and in mammalian cells, and possibility to be inserted inside of tagged proteins. Spectral properties of the CBCR-derived NIR FP enable its application as an efficient fluorescence resonance energy transfer (FRET) donor for a red-shifted BphP-derived NIR FP acceptor and engineering of fully-NIR kinase biosensors. Cross-talk-free use of the NIR biosensors with blue-light-activatable optogenetic kinase inhibitors in the same cells demonstrates the applicability of the CBCR-based NIR FP in all-optical techniques.

## Results

### Engineering of the CBCR GAF domain into BV-binding FP

To choose a template for engineering of BV-binding CBCR-based NIR FP, we evaluated GAF domains from ten different CBCRs (Supplementary Fig. 1a). To facilitate protein production in mammalian cells, we first *codon-optimized* the CBCR genes for mammalian cell expression. To facilitate BV binding, we then introduced Leu residues at the position corresponding to Leu337 in AM1_1557g2, which was shown being important for the BV attachment^18,20^. To reduce size of the CBCR GAF domains, we next removed the N-terminal α1-helix, which does not participate in the formation of the tetrapyrrole-binding pocket^24^.

**Fig. 1:**
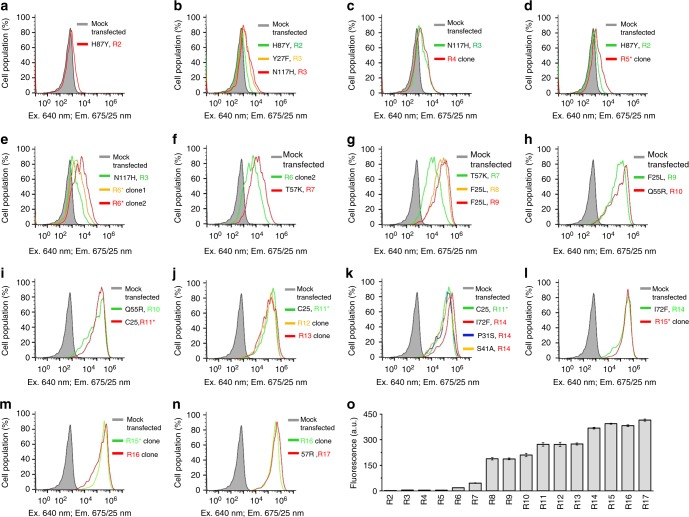
Molecular engineering of miRFP670nano. **a**–**n** Comparison of clones selected on each round of selection in HeLa cells. The main mutations are indicated. **o** Quantification of the data represented in **a**–**n**. Mean NIR fluorescence intensity was normalized to mean green fluorescence intensity of co-expressed EGFP and to mean fluorescence intensity of mock-transfected cells. Error bars, s.d. (*n* = 3; transfection experiments)

These CBCRs were co-expressed in *E. coli* with heme oxygenase for BV production; however, they exhibited very weak or no fluorescence. Interestingly, an NpR3784 GAF domain substantially outperformed the GAF domains of other CBCRs, including AM1_1557g2 and AM1_1870g3 that earlier were shown to bind BV (Supplementary Fig. 1b, c). We subjected the NpR3784 GAF domain to several rounds of random mutagenesis, followed by saturating mutagenesis of the identified residues (Supplementary Fig. 2). After each round, we tested the best clones in mammalian cells and selected for the next molecular evolution only those which exhibited the high fluorescence brightness in both bacteria and mammalian cells (Fig. 1). Totally, 17 rounds of the directed molecular evolution resulted in an NIR FP variant, termed miRFP670nano, consisting of 147 amino acid residues (17 kDa) and bearing 18 substitutions (numbering follows that for miRFP670nano sequence) relative to wild-type GAF from NpR3784: V7M, F25C, M26V, Y27F, P31E, S41A, A48S, N51K, Q55R, T57R, I72Y, G82N, H87Y, N99I, N117H, C119L, L136Q, and Q139V (Supplementary Fig. 2).

### Characterization of miRFP670nano protein in vitro

**Fig. 2:**
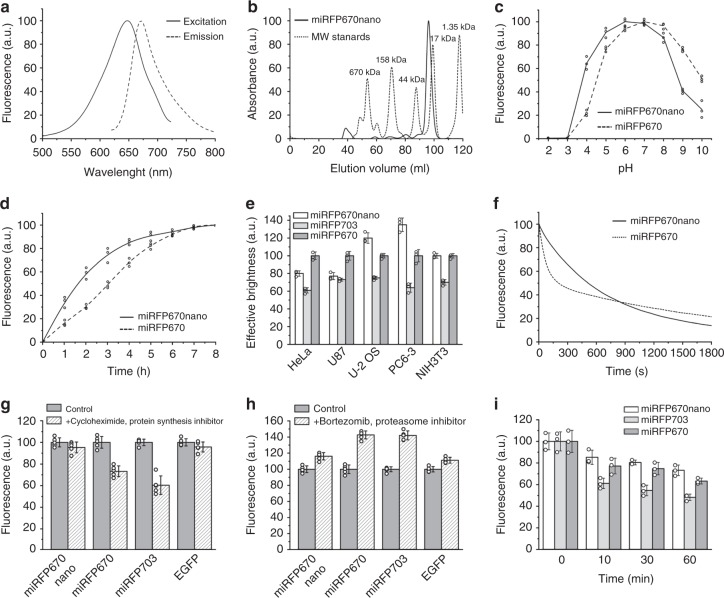
Characterization of miRFP670nano. **a** Fluorescence excitation and emission spectra of miRFP670nano. **b** Size-exclusion chromatography of miRFP670nano at concentration 10 mg ml^−1^ and indicated molecular weight standards. miRFP670nano with polyhistidine tag and linker runs as a monomer with the apparent molecular weight of 18.8 kDa. **c** pH dependencies of NIR fluorescence for miRFP670nano and miRFP670. **d** Kinetics of miRFP670nano and miRFP670 maturation. Time “0” corresponds to the beginning of the 1-h-long pulse-chase induction of the protein expression in bacteria. **e** Effective (cellular) brightness of miRFP670nano, miRFP703, and miRFP670 in mammalian cells. Live HeLa, U87, U-2 OS, PC6-3, and NIH3T3 cells were transiently transfected with miRFP670nano, miRFP703, or miRFP670. Fluorescence was analyzed by flow cytometry 72 h after transfection. NIR fluorescence intensity was normalized to that of co-transfected EGFP (to account for differences in transfection efficiency), to excitation efficiency of each NIR FP by 640 nm laser, and to emission spectrum of each FP in the emission filter. Effective brightness of miRFP670 was assumed to 100% for each cell line. Error bars, s.d. (*n* = 3; transfection experiments). **f** Photobleaching of miRFP670nano and miRFP670 in live HeLa cells. **g** Mean fluorescence intensity of HeLa cells transiently transfected with miRFP670nano, miRFP703, miRFP670, and EGFP before and after 4 h of incubation with 20 µg ml^−1^ cycloheximide. Error bars, s.d. (*n* = 5; transfection experiments). **h** Mean fluorescence intensity of HeLa cells transiently transfected with miRFP670nano, miRFP703, miRFP670, and EGFP before and after 4 h of incubation with 10 µM bortezomib. Error bars, s.d. (*n* = 5; transfection experiments). **i** Tolerance of miRFP670nano to fixation in paraformaldehyde. HeLa cells transfected with miRFP670nano, miRFP670, and miRFP703 were incubated with 4% paraformaldehyde for 10–60 min. The fluorescence of cells treated with paraformaldehyde was normalized to fluorescence of non-fixed cells. Error bars, s.d. (*n* = 3; transfection experiments)

miRFP670nano exhibited fluorescence excitation and emission maxima at 645 and 670 nm, respectively, which were close to those observed for blue-shifted two-domain BphP-based NIR FPs, like miRFP670 (Fig. 2a and Table 1). Absorbance of miRFP670nano had a minor peak at 390 nm corresponding to the Soret band, characteristic for tetrapyrrole-binding proteins, and a major peak at 645 nm, suggestive of the efficient BV incorporation (Supplementary Fig. 3a). miRFP670nano exhibited monomeric behavior in size-exclusion chromatography at high concentration of 10 mg ml^−1^ (Fig. 2b and Supplementary Fig. 3b). Notably, with fluorescence quantum yield of 10.8% and extinction coefficient of 95,000 M^−1^ cm^−1^, molecular brightness (a product of molar extinction coefficient and quantum yield) of miRFP670nano exceeded that of the most of BphP-based NIR FPs (Table 1).

**Table 1:** Properties of currently available monomeric NIR FPs designed from various bacterial photoreceptors

NIR FP	Parental bacterial photoreceptor	Ex (nm)	Em (nm)	Extinction coefficient (M^−1^cm^−1^)	Quantum yield (%)	Molecular brightness vs. miRFP670 (%)	Oligomeric state	p*K*a	Photostability in HeLa cells, *t*_1/2_ (s)	Brightness in mammalian cells vs miRFP670 (%)^a^	Ref.
miRFP670 nano	NpR3784 cyanobacteriochrome	645	670	95,000	10.8	84	Monomer	3.7	505	100^b^	This work
miRFP670	RpBphP1 bacterial phytochrome	642	670	87,400	14.0	100	Monomer	4.5	183	100	^3^
miRFP703		674	703	90,900	8.6	64	Monomer	4.5	394	61	
miRFP709		683	709	78,400	5.4	35	Monomer	4.5	192	42	
miRFP720	RpBphP2 bacterial phytochrome	702	720	98,000	6.1	49	Monomer	4.5	510	160	^5^
mIFP	BrBphP bacterial phytochrome	683	704	82,000	8.4	56	Monomer	4.5	54	26	^4, 8^
smURFP	TeAPCα allophycocyanin	642	670	180,000^c^	18.0	265^c^	Dimer	–	570	1	^6, 8^
BDFP1.5	ApcF allophycocyanin	688	711	74,000	5.0	30	Monomer	2.0	1310^d^	0.5^d^	^7^

^a^Unless otherwise stated, it is determined as effective NIR fluorescence in live HeLa cells 72 h after transfection with no supply of exogenous BV and after normalization to fluorescence of co-transfected EGFP

^b^Effective brightness of miRFP670nano in various mammalian cell lines is compared in Fig. 2e

^c^Determined for a dimer of smURFP molecules

^d^Based on the comparison with smURFP in HEK293 cells in ref. ^7^

miRFP670nano had substantially higher protein stability than BphP-based NIR FPs. Studies of a pH dependence revealed that miRFP670nano fluorescence is stable between pH 4.0 and 8.0, with p*K*a = 3.7, which was notably acid-shifted than for BphP-derived NIR FPs, having p*K*a = 4.5 (Fig. 2c and Table 1). Moreover, after 24 h incubation in 3.0 M guanidine hydrochloride miRFP670nano retained ~80% of its fluorescence, whereas miRFP670 and miRFP703 were stable up to 1.5 M guanidine hydrochloride concentration only (Supplementary Fig. 4). Likely, the compact and tight structure enhanced the miRFP670nano resistance to denaturating conditions.

miRFP670nano maturation had a half-time of ~100 min (Fig. 2d), which was 1.8-fold faster than for spectrally similar two-domain miRFP670, suggesting that the single-domain structure and the absence of the characteristic for all BphP-based NIR FPs figure-of-eight knot structure accelerated the miRFP670nano folding.

### Performance of miRFP670nano in mammalian cells

miRFP670nano efficiently binds endogenous BV in mammalian cells. The cellular (a.k.a. effective) brightness of miRFP670nano was comparable to that to miRFP670 and exceeded that of miRFP703 in all tested mammalian cells (Fig. 2e). The high effective brightness in the absence of exogenous BV in mammalian cells is an essential advantage of BphP-based NIR FPs over APC-derived FPs (Table 1)^3,7,8,16,17^. While BV is the major chromophore for BphPs, it is not the case for CBCRs for which PCB is the primary tetrapyrrole co-factor, emphasizing the efficiency of the applied molecular evolution resulted in miRFP670nano.

In mammalian cells miRFP670nano exhibited 2.8- and 1.3-fold higher photostability than miRFP670 and miRFP703, respectively (Fig. 2f, Table 1). miRFP670nano also exhibited low cytotoxicity (Supplementary Fig. 5). The high photostability, low cytotoxicity, and high effective brightness make miRFP670nano a favorable NIR FP for imaging of long-term cellular events.

miRFP670nano is highly stable in mammalian cells. Protein degradation analysis showed that after 4 h incubation with a protein synthesis inhibitor cycloheximide miRFP670nano-expressing cells retained ~95% of their fluorescence (Fig. 2g). Similar cellular stability was observed for enhanced green fluorescent protein (EGFP). In contrast, cells expressing miRFP670 or miRFP703 retained only ~70 and 60% of fluorescence, respectively. Furthermore, incubation with bortezomib, an inhibitor of proteasome-dependent protein degradation, just slightly increased brightness of the miRFP670nano- and EGFP-expressing cells (16 and 11%, respectively) (Fig. 2h). On the contrary, the cellular brightness of BphP-based FPs was increased more than 40% after inhibition of proteosomal degradation. Moreover, a comparison of the number of fluorescent cells 48 and 120 h after transfection for all these FPs confirmed the high miRFP670nano cellular stability (Supplementary Fig. 6). While overall number of miRFP670- and miRFP703-expressing cells decreased more than twice 120 h after transfection, the number of miRFP670nano-expressing cells decreased ~10% only, which was similar to those with EGFP.

Live-cell imaging allows monitoring of dynamic events, but some studies require cell fixation. We compared tolerance of miRFP670nano and BphP-based miRFP670 and miRFP703 to fixation with 4% paraformaldehyde. Again, the miRFP670nano-transfected cells demonstrated the highest stability and retained more than 80% of fluorescence after 30 min fixation (Fig. 2i).

Overall, likely due to the compact and robust protein fold CBCR-derived miRFP670nano exhibits the high cellular brightness and significantly enhanced protein stability in vitro and in mammalian cells.

### Structural basis of miRFP670nano properties

**Fig. 3:**
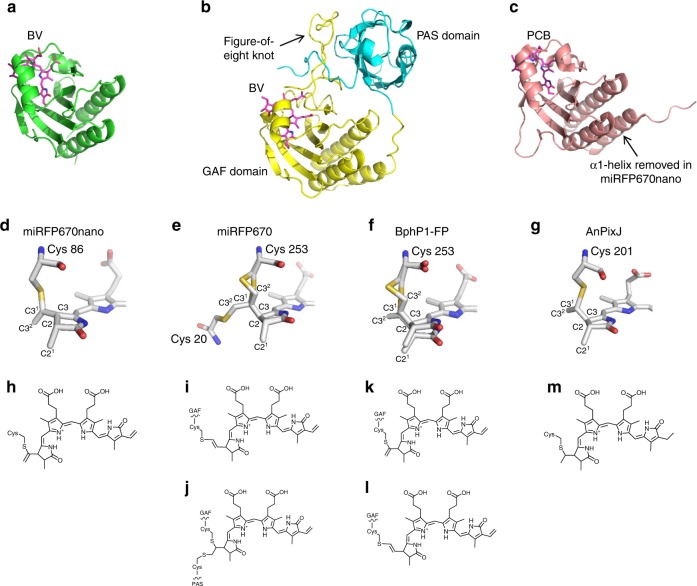
Comparison of miRFP670nano, miRFP670, BphP1-FP, and AnPixJ structures and chromophores. **a**–**c** Overall structures of **a** miRFP670nano, **b** miRFP670 (PDB ID: 5VIV), BphP1-FP (PDB ID: 4XTQ), and **c** AnPixJ (PDB ID: 3W2Z). The BV and PCB chromophores are in magenta. α1-Helix removed in miRFP670nano is indicated in AnPixJ structure. The PAS and GAF domains of miRFP670 are in cyan and yellow, respectively, and the figure-of-eight knot is indicated. Because of the very similar structures of miRFP670 and BphP1-FP, only the former one is shown. **d**–**g** Chromophores (rings A and B only) bound to Cys residues in **d** miRFP670nano, **e** miRFP670, **f** BphP1-FP, and **g** AnPixJ. Carbon, nitrogen, oxygen, and sulfur atoms are in white, blue, red, and yellow, respectively. Single chromophore species are observed in miRFP670nano and AnPixJ only. Two BV chromophore species are observed in miRFP670 and BphP1-FP. **h**–**m** Chemical formulas of the chromophores in **h** miRFP670nano, **i**, **j** miRFP670, **k**, **l** BphP1-FP, and **m** AnPixJ. In miRFP670nano, the BV chromophore (**h**) is bound to the Cys86 residue via the C3^1^ atom. In miRFP670 the BV chromophore (**i**) is bound via the C3^2^ atom to the Cys253 in the GAF domain, and the BV chromophore (**j**) is bound via the C3^1^ atom to Cys253 in the GAF domain and also via the C3^2^ atom to Cys20 in the PAS domain. In BphP1-FP the BV chromophore (**k**) is bound via the C3^1^ atom to Cys253 in the GAF domain, and the BV chromophore (**l**) is bound via the C3^2^ atom to Cys253 residue in the GAF domain. In AnPixJ the PCB chromophore (**m**) is bound to the Cys201 residues via the C3^1^ atom

To reveal the structural basis of the protein stability, brightness, and specificity to BV chromophore, we determined the crystal structure of miRFP670nano at 1.95 Å resolution (Fig. 3 and Supplementary Fig. 7). miRFP670nano adopts a GAF-domain fold, but with the N and C termini located in the spatial proximity (Fig. 3a and Supplementary Fig. 7a). The GAF domain of BphPs has the similar fold; however, it is topologically linked to the adjacent PAS domain via a loop in the figure-of-eight knot (Fig. 3b). The closest available structure of the GAF domain of CBCR is a structure of putative phototaxis regulator PixJ of *Anabaena* sp. *PCC 7120*, AnPixJ, in the red-absorbing state^24^ (Fig. 3c).

BV is covalently attached by a thioether bond between the conserved for CBCRs Cys86 residue and the C3^1^ atom of the ring A (Fig. 3d, h and Supplementary Fig. 7b), similar to the native CBCR’s PCB chromophore (Fig. 3g, m), but having a double bond between C3^1^ = C3^2^. This mode of the BV binding is different than in natural BphPs and red-shifted BphP-derived NIR FPs in which BV is attached via the C3^2^ atom of the ring A to a conserved Cys in the PAS domain. Recently, however, the unusual covalent binding of BV to Cys in the GAF domain was described for blue-shifted NIR FPs, such as miRFP670 and BphP1-FP, containing the engineered Cys residue in the GAF domain^21,22,25^. While only one BV chromophore type is detected in the crystal structure of miRFP670nano (Fig. 3d), the blue-shifted BphP-based NIR FPs have two different chromophore types^21,22^ (Fig. 3e, f), resulting in two distinct protein species present in miRFP670, as well as in BphP1-FP. miRFP670nano chromophore has the same number of conjugated double bonds as the chromophores in blue-shifted BphP-based NIR FPs (Fig. 3h–l) that explains the similarity of spectra for these three NIR FPs^21^.

Immediate chromophore environment is critical for BV binding and fluorescence of miRFP670nano. In the chromophore-binding pocket, BV is stabilized by eight hydrogen bonds with D56, Y67, T84, R71, and H117, π–π stacking with Y87, and T-stacking with F59 (Supplementary Fig. 7c, d). A pyrrole water, a proton donor providing excited-state proton transfer in BphP-derived NIR FPs and natural BphPs, is absent in miRFP670nano, similar to CBCR AnPixJ^24^. Its role is likely played by the side chain of D56, which forms H-bonds with pyrrole nitrogens of the rings A, B, and C^24^.

Of the 18 amino acid substitutions introduced into parental NpR3784g (Supplementary Fig. 7e), F25C, Y27F, H87Y, N99I, and N117H are located within 3.6 Å of the chromophore and either directly stabilize it or provide for it favorable accommodation. One of the important substitutions, N117H makes a strong H-bond with the ring D, which is absent in NpR3784g, thus preventing rotation of this ring and non-radiative energy dissipation via photoswitching. Another critical mutation is H87Y, which introduced a perfect parallel π–π stacking with BV. F25C makes additional space in the chromophore-binding pocket, possibly enhancing BV accommodation. T57R substitution introduced a flexible positively charged residue near the chromophore binding site, enabling electrostatic attraction of BV and its additional shielding from solvent. Substitutions Y27F and N99I increased the hydrophobicity of the chromophore environment.

Such a favorable chromophore binding pocket within a compact single GAF domain fold should make miRFP670nano a robust probe for various applications.

### Performance of miRFP670nano as protein fusion tag

**Fig. 4:**
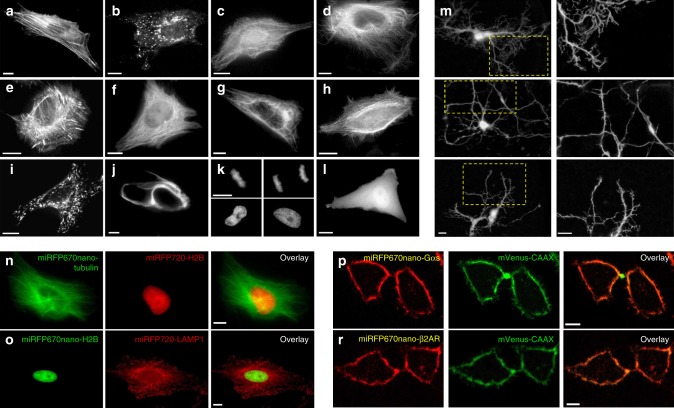
miRFP670nano fusions imaged using epifluorescence microscopy. Live HeLa cells transfected with the miRFP670nano N- and C-terminal fusion constructs. The C-terminal fusions are **a** actin; **b** vesicular protein clathrin; **c** myosin; **d** α-tubulin. The N-terminal fusions are **e** α-actinin; **f** microtubules-binding EB3; **g** keratin; **h** actin-binding LifeAct; **i** lysosomal membrane glycoprotein LAMP1; **j** vimentin; **k** histone H2B. **l** Cells expressing untagged miRFP670nano. **m** Dissociated rat cortical neurons transfected with miRFP670nano encoding plasmid at 3 days in vitro (DIV 3). Neurons were imaged 48 h after transfection. Left images are zoom-in of the indicated areas of the right images. **n** Two-color images of cells co-expressing α-tubulin tagged with miRFP670nano and H2B tagged with miRFP720. **o** Two-color images of cells co-expressing LAMP1 tagged with miRFP720 and H2B tagged with miRFP670nano. **p** miRFP670nano internally inserted between the helical and GTPase domains of the G-protein α subunit (Gαs). **r** miRFP670nano internally inserted into the intracellular loop 3 of the β2 adrenergic receptor (β2AR). mVenus with membrane targeting CAAX motif was used for membrane visualization. Scale bars, 10 μm

To test performance of miRFP670nano as fluorescent probe for labeling of intracellular structures, we constructed several miRFP670nano N- and C-terminal protein fusions. In live mammalian cells these fusions exhibited proper localization, including the fusions associated with or forming filaments (Fig. 4). miRFP670nano fusion with histone 2B localized properly in different phases of mitosis and did not affect cell division (Fig. 4k). Cell images showed homogenous distribution of miRFP670nano and absence of intracellular aggregates (Fig. 4l).

In number of cases, placing of a FP tag at the termini of proteins affects their function or leads to incorrect localization^26^. Such proteins can be labeled with FP inserted in a middle of the sequence as an internal tag. For this, FP should have good folding properties and its N- and C-termini located close to each other, like in miRFP670nano (Fig. 3a and Supplementary Fig. 7a).

To evaluate miRFP670nano as an internal tag, we constructed internally labeled G protein α-subunit (Gαs) and β2-adrenergic receptor (β2AR) in which miRFP670nano or miRFP670 were inserted between the helical and GTPase domains of Gαs and into intracellular loop 3 of β2AR^27,28^. Both miRFP670nano internal fusions demonstrated perfect membrane localization, co-localizing with mVenus containing a CAAX-motif for membrane targeting. In contrast, the internal fusion constructs with two-domain miRFP670 did not exhibit membrane localization and formed aggregates (Fig. 4p, r and Supplementary Fig. 8a–d). Most likely the complex structural organization of BphP-derived miRFP670 interfered with folding of internally tagged Gαs and β2AR. Notably, unlike BphPs, GAF domains of CBCRs are often found as modular components of complex signaling proteins^15^, suggesting that miRFP670nano has naturally optimized structure for flexible design of fusion constructs.

We next evaluated applicability of miRFP670nano for imaging of primary cell cultures, such as neurons. Primary rat cortical neurons transfected with miRFP670nano exhibited bright homogenous fluorescence without supplying exogenous BV (Fig. 4m).

To evaluate miRFP670nano in two-color NIR imaging with monomeric BphP-derived red-shifted miRFP720^5^, we imaged HeLa cells co-expressing different miRFP670nano and miRFP720 fusions (Fig. 4n, o). All fusions had proper localization and clear separation of miRFP670nano and miRFP720 fluorescence signals. Notably, endogenous BV concentration was sufficient to provide bright fluorescence to both NIR FPs co-expressed in the same cells.

### NIR FRET biosensors of PKA and JNK kinases

The high photostability, small size, and relatively high quantum yield make miRFP670nano a promising FRET donor for red-shifted miRFP720^5^. To evaluate this FRET pair, we fused miRFP670nano and miRFP720 via linker with a cleavage site for caspase-3, the key protease in apoptosis (Supplementary Fig. 9a). Upon apoptosis induced by staurosporine, we observed ~1.65-fold decrease in the FRET/miRFP670nano fluorescence ratio detected at 725 nm/667 nm in HeLa cells transfected with the miRFP670nano-miRFP720 caspase-3 reporter (Supplementary Fig. 9b-e). These results suggested that miRFP670nano and miRFP720 can be successfully used to design fully NIR FRET biosensors.

**Fig. 5:**
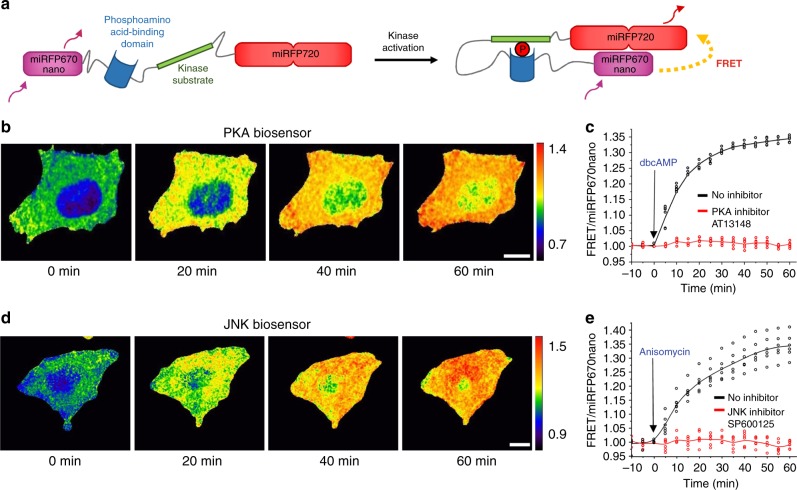
NIR biosensors for detection of PKA and JNK kinase activities. **a** Schematic representation of miRFP670nano-miRFP720-based NIR FRET biosensor for kinase activity. **b** Time-lapse FRET/miRFP670nano ratio images of HeLa cell expressing NIR PKA biosensor stimulated with 1 mM dbcAMP and visualized using pseudocolor. **c** FRET/miRFP670nano ratio time courses of HeLa cells expressing PKA biosensor stimulated with dbcAMP in the presence (red) and absence (black) of chemical PKA inhibitor, AT13148 (*n* = 3 independent experiments). **d** Time-lapse FRET/miRFP670nano ratio images of HeLa cell expressing NIR JNK biosensor stimulated with 1 μg ml^−1^ anisomycin and visualized using pseudocolor. **e** FRET/miRFP670nano ratio time courses of HeLa cells expressing JNK biosensor stimulated with anisomycin in the presence (red) and absence (black) of chemical JNK inhibitor, SP600125 (*n* = 3 independent experiments). In **b**–**e** the miRFP670nano and FRET fluorescence signals were detected at 667 and 725 nm, respectively. Scale bars, 10 μm

We next constructed biosensors for detection of protein kinase A (PKA) and c-Jun N-terminal kinase (JNK) activities^29^. PKA is one of the key effectors of cAMP-mediated signaling pathway, while JNK regulates cellular responses to diverse environmental stress signals and inflammatory cytokines^30^. The NIR biosensors consisted of a miRFP670nano donor, a phosphoamino acid-binding domain, a consensus peptide sequence of kinases substrates, and a miRFP720 acceptor (Fig. 5a). Phosphorylation of the substrate peptide by activated kinases leads to a conformation rearrangement of the biosensor and an increase of FRET between donor and acceptor.

Stimulation of HeLa cells stably expressing NIR PKA biosensor with 1 mM dibutyryl cyclic adenosine monophosphate (dbcAMP) led to a fast increase in the FRET/miRFP670nano fluorescence ratio, which reached ~33% in 1 h. The response was not detected in the presence of PKA inhibitor AT13148 (Fig. 5b, c). Treatment of HeLa cells expressing the NIR JNK biosensor with 1 μg ml^−1^ anisomycin, a JNK agonist^31^, led to an increase of the FRET/miRFP670nano fluorescence ratio with typical for JNK kinetics^32^, which reached ~35% in 1 h. Incubation with JNK inhibitor SP600125 prevented the response to anisomycin (Fig. 5d, e). Both NIR biosensors exhibited the high dynamic range, similar to that for the PKA and JNK biosensors based on the ECFP-Venus and ECFP-Citrine pairs^32,33^.

### Spectral multiplexing of NIR biosensors

**Fig. 6:**
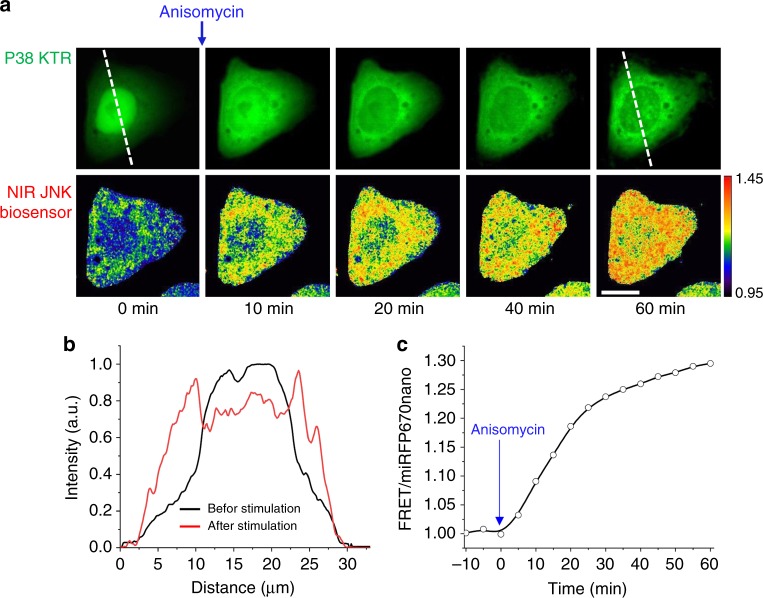
HeLa cell stably expressing NIR JNK biosensor co-transfected with p38 kinase translocation reporter (p38 KTR). **a** p38 KTR-EGFP translocation (top row) and FRET/miRFP670nano ratio changes (bottom row) upon stimulation with 1 μg ml^−1^ anisomycin. Dashed line marks the region used for profile plotting. FRET/miRFP670nano ratio images are visualized using intensity pseudocolor. Scale bar, 10 µm. **b** Intensity profiles of p38 KTR-EGFP fluorescence before and after stimulation with anisomycin. **c** Kinetics of FRET/miRFP670nano ratio upon stimulation with anisomycin. The miRFP670nano and FRET fluorescence signals were detected at 667 and 725 nm, respectively

Important advantage of fully NIR biosensors is their spectral compatibility with GFP-like FPs and common optogenetic tools activatable with blue light. We co-expressed NIR JNK biosensor and EGFP-based p38 kinase translocation reporter (p38 KTR)^34^ in HeLa cells. Similarly to JNK, p38 kinase is activated by stress signals and inflammatory cytokines^30,31^. After treatment of cells with anisomycin, which induces activation of both kinases^31^, we observed response of both biosensors, NIR JNK and p38 KTR (Fig. 6).

**Fig. 7:**
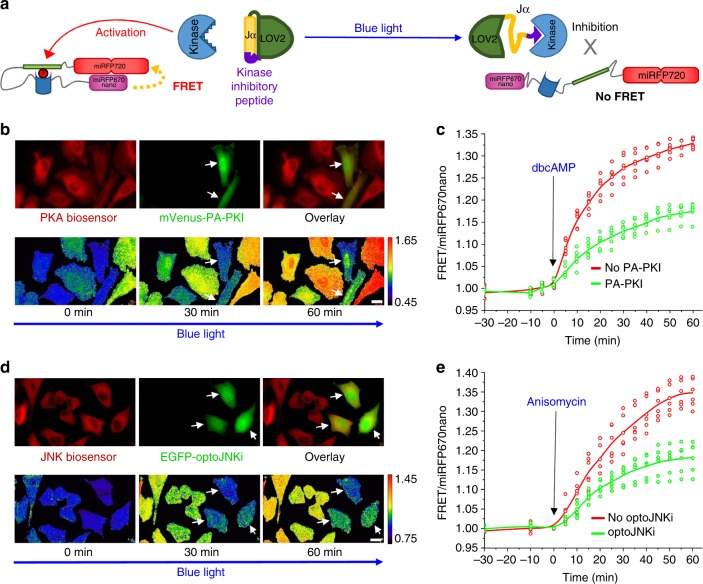
Multiplexing of NIR PKA and JNK biosensors with optogenetic kinase inhibitors. **a** Schematic representation of LOV2-domain-based blue-light-regulatable kinase inhibitor in combination with respective fully-NIR kinase biosensor. Upon illumination with blue light, the Jα helix of LOV2 unfolds, resulting in uncaging of a peptide, which inhibits kinase. **b** HeLa cells stably expressing NIR PKA biosensor co-transfected with optogenetic PKA inhibitor, PA-PKI, tagged with mVenus (top row). Upon simultaneous 460 nm illumination and stimulation with 1 mM dbcAMP, the changes in FRET/miRFP670nano ratio are shown in pseudocolor (bottom row). **c** FRET/miRFP670nano ratio time courses of HeLa cells expressing NIR PKA biosensor only (red) or NIR PKA biosensor with PA-PKI (green) upon simultaneous 460 nm illumination and stimulation with 1 mM dbcAMP (*n* = 3 independent experiments). **d** HeLa cells stably expressing JNK biosensor co-transfected with optogenetic JNK inhibitor, optoJNKi, tagged with EGFP (top row). Upon simultaneous 460 nm illumination and stimulation with 1 μg ml^−1^ anisomycin, the changes in FRET/miRFP670nano ratio are shown in pseudocolor (bottom row). **e** FRET/miRFP670nano ratio time courses of HeLa cells expressing NIR JNK biosensor only (red) or NIR JNK biosensor with optoJNKi (green) upon simultaneous 460 nm illumination and stimulation with anisomycin (*n* = 3 independent experiments). White arrows indicate cells expressing optogenetic regulators. In **b**–**e** the miRFP670nano and FRET fluorescence signals were detected at 667 and 725 nm, respectively. Scale bars, 10 μm

While the combination of several biosensors enables monitoring of several cell processes, a combination of biosensors with optogenetic tools should allow simultaneous detection and regulation of the processes. This is a powerful all-optical approach to study cell signaling in native environment. Recently, a blue-light-controlled optogenetic JNK inhibitor (optoJNKi) and a photoactivatable PKA inhibitor (PA-PKI), based on the LOV2 domain from *Avena sativa* phototropin 1, were developed^35,36^. In these optogenetic tools, PKA or JNK inhibitory peptides are fused to a Jα helix of the LOV2 domain (Fig. 7a). In darkness, the peptides are sterically blocked from kinase interaction, whereas blue light leads to unfolding of the Jα helix, uncaging the peptides and, consequently, to kinase inhibition^35,36^. Absorbance spectrum of LOV2 domain and excitation spectra of miRFP670nano donor and miRFP720 acceptor have minimal overlap (Supplementary Fig. 10).

To evaluate compatibility of optoJNKi and PA-PKI with NIR biosensors, we transfected HeLa cells stably expressing JNK or PKA biosensors with the respective optogenetic inhibitors. Cells transfected with the optogenetic tools responded to stimuli similarly to the cells expressing the biosensors only (Supplementary Fig. 11 and 12). However, under blue light cells with the optogenetic constructs exhibited the substantial decrease in response to the stimuli (Fig. 7b–e). This demonstrated that the NIR JNK and PKA biosensors can be efficiently spectrally multiplexed with optogenetic tools in the same cells.

### Characterization of miRFP670nano in vivo

**Fig. 8:**
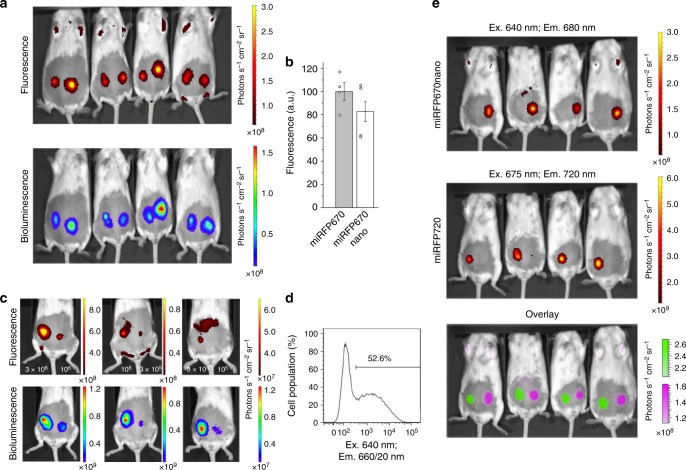
Characterization of miRFP670nano in vivo. **a** Comparison of miRFP670nano with miRFP670 in vivo. Fluorescence (top row) and bioluminescence (bottom row) images of living mice injected with 3 × 10^6^ HeLa cells expressing miRFP670 (left) and miRFP670nano (right). Cells were co-transfected with Rluc8 (miRFPs:Rluc8 plasmid ratio is 10:1). The fluorescence images were obtained with excitation at 640 nm and emission at 680 nm using IVIS Spectrum instrument 72 h after cell transfection. **b** Brightness of injected HeLa cells expressing miRFP670 or miRFP670nano as shown in **a**. Mean fluorescence intensity was normalized to mean bioluminescence intensity. Error bars, s.d. (*n* = 3 experiments). **c** Minimal amount of detectable miRFP670nano cells. Fluorescence (top row) and bioluminescence (bottom row) images of living mice injected with various quantity of HeLa cells expressing miRFP670nano. Left mouse was injected with 3 × 10^6^ (left) and 10^6^ (right) cells; middle mouse was injected with 10^6^ (left) and 3 × 10^5^ (right) cells; right mouse was injected with 3 × 10^5^ (left) and 10^5^ (right) cells. Cells were co-transfected with Rluc8 (miRFPs:Rluc8 plasmid ratio is 10:1). The fluorescence images were obtained with excitation at 640 nm and emission at 680 nm 72 h after cell transfection. **d** Transfection efficiency of injected HeLa cells obtained by FACS analysis. **e** Two-color imaging of miRFP670nano and miRFP720 in vivo. Fluorescence images of living mice injected with 3 × 10^6^ HeLa cells expressing miRFP670nano (top row) and miRFP720 (middle raw) and its overlay (bottom raw) are shown. The fluorescence images were obtained with excitation at 640 nm and emission at 680 nm for miRFP670nano and with excitation at 675 nm and emission at 720 nm for miRFP720 72 h after cell transfection

To compare miRFP670nano with miRFP670 in in vivo imaging, we injected miRFP670nano- or miRFP670-expressing HeLa cells co-transfected with RLuc8 luciferase in mammary glands of mice. The miRFP670 and miRFP670nano fluorescence signals were normalized to Rluc8 bioluminescence to account for transfection efficiency. We found that in vivo brightness of miRFP670nano cells was comparable to that of miRFP670 cells (Fig. 8a, b). We then estimated the minimal detectable quantity of cells expressing miRFP670nano. For this, we injected in mammary glands various amounts of transiently transfected cells and found that we were able to detect ~1.5 × 10^5^ fluorescent cells (Fig. 8c, d). We next tested miRFP670nano in two-color whole-body imaging. Cells transfected with either miRFP670nano or miRFP720 were well spectrally distinguished in mice using two channels with ex./em. at 640 nm/680 nm and 675 nm/720 nm, respectively (Fig. 8e). Overall, these in vivo results showed that miRFP670nano performs well in whole-body imaging and can be used in combination with red-shifted NIR FPs.

## Discussion

By applying 17 rounds of molecular evolution to the GAF domain of PCB-binding NpR3784 CBCR, we have developed the first CBCR-based NIR FP, which efficiently binds endogenous BV and fluoresces in various mammalian cells (Figs. 2e, 4). With molecular weight of only 17 kDa, miRFP670nano is the smallest monomeric NIR FP that fluoresces in mammalian cells as bright as twice bigger state-of-art two-domain NIR FPs.

We step-by-step compared miRFP670nano with two spectrally closest monomeric BphP-based NIR FPs, miRFP670, and miRFP703 (Table 1). The effective brightness of miRFP670nano in cells is similar to that of miRFP670 and exceeds that of miRFP703 (Fig. 2e). The only example of a single-domain BphP-based FP, monomeric GAF-FP^37^, has more than 30-fold lower cellular brightness, and all APC-derived FPs have more than 80-fold lower cellular brightness than miRFP670nano^6–8^ (Table 1).

Comparing to BphP-based NIR FPs, miRFP670nano is characterized by high stability to acidic environment, denaturation conditions, cell fixation, and degradation in mammalian cells (Fig. 2c, g, h, i and Supplementary Fig. 4 and 6). Compact protein fold with N and C termini in a close proximity allows the use of miRFP670nano not only as a protein terminal tag but also as an insertion inside the loops of a protein of interest, as demonstrated for Gαs and β2AR (Fig. 4p, r and Supplementary Fig. 8). In contrast, two-domain BphP-derived NIR FPs, which have the figure-of-eight knot in their structure and distant termini, are not suitable for internal tagging.

The crystal structure of miRFP670nano allows to visualize the chromophore and its immediate environment optimized during molecular evolution for BV binding and fluorescence (Supplementary Fig. 7). Bound to the conserved Cys86 via its C3^1^ atom, BV forms a chromophore, which lacks a double bond between C2 and C3, but has a double bond between C3^1^ and C3^2^. The number of conjugated double bonds in this chromophore is the same as in the chromophores of blue-shifted BphP-derived NIR FPs that explains their spectral similarity (Fig. 3h–l). However, the BphP-derived NIR FPs have two different chromophore types (Fig. 3e, f), which leads to heterogeneity of the protein species and, consequently, affects their properties. Likely, the presence of two protein species in miRFP670 explains its slightly sigmoidal maturation (Fig. 2d), bi-exponential photobleaching (Fig. 2f), and wider spread of denaturation dependence (Supplementary Fig. 4). The chromophore homogeneity is another important advantage of CBCR-based miRFP670nano over blue-shifted BphP-based NIR FPs.

Relatively high quantum yield of miRFP670nano and good overlap of its emission with miRFP720 excitation make miRFP670nano a favorable FRET donor for red-shifted NIR FPs. That was demonstrated by the development of efficient fully-NIR biosensors of PKA and JNK activities (Fig. 5). JNK is key transducer of exogenous stress signals and is involved in regulation of a number of physiological and pathological processes including apoptosis, proliferation, embryonic development, and inflammation. PKA mediates signals of G protein-coupled receptors and regulates a plethora of downstream effectors involved in key cellular processes. The developed NIR PKA and JNK biosensors enable multiplexing with blue-green optogenetic tools for probing and monitoring of multiple cell processes for better understanding of mechanisms that mediate regulation and specificity of PKA and JNK kinases. Simultaneous detection and light control of the PKA and JNK activities using the fully-NIR miRFP670nano-miRFP720-based biosensors and the respective blue-light-activatable kinase regulators (Fig. 7) demonstrated the wide applicability of miRFP670nano and miRFP670nano-miRFP720 FRET pair in non-invasive all-optical assays in single cells and in vivo.

NIR fluorescence makes miRFP670nano a useful probe not only for crosstalk-free spectral multiplexing in microscopy but also for deep-tissue imaging. In mice, miRFP670nano performed similarly to miRFP670 and could be used in multicolor tissue labeling with red-shifted NIR FP (Fig. 8).

To date, the large number of CBCRs has been cloned. Unlike natural BphPs, different subclasses of CBCRs exhibit the remarkable spectral diversity, sensing light from UV to NIR spectral ranges^1^. The CBCR spectral tuning is mainly associated with the characteristic amino acid motifs^38^, frequently containing Cys residues able to form thioether bonds with different carbon atoms of the PCB chromophore, hence affecting the degree of its electron conjugation. Directed mutagenesis allows changing of CBCR absorbing spectra; for example, a red/green NpR6012g4 GAF domain was converted to a blue/green variant with three amino acid substitutions only^39^. We hypothesize that CBCRs can be used as a source of the whole new class of small and stable BV-binding FPs with spectral variety from UV to NIR. The structure and protein engineering strategy described in this work for NpR3784-derived NIR FP miRFP670nano should open the door to future BV-binding FPs with new photophysical and biochemical properties.

## Methods

### Mutagenesis and directed molecular evolution

The CBCR GAF genes were synthesized by GenScript Company. The DNA sequences were optimized with OptimumGene algorithm (GenSript), taking into account the codon usage bias (human cells), GC (guanine-cytosine) content, CpG dinucleotides content, messenger RNA secondary structure, and other parameters. For expression in bacteria, DNA sequences encoding the GAF domains were cloned into pBAD/His-B vector (Life Technologies/Invitrogen) by *Kpn*I/*Eco*RI sites. BV synthesis in bacteria was facilitated by co-transformation with a pWA23h plasmid encoding heme oxygenase from *Bradyrhizobium ORS278* (hmuO) under the rhamnose promoter^17,40^. LMG194 host cells (Invitrogen) were used for protein expression.

All oligonucleotide primers for polymerase chain reaction (PCR) were purchased from Biomers (Supplementary Table 1). For simultaneous site-specific mutagenesis at several positions, an overlap-extension approach was applied. Random mutagenesis was performed with GeneMorph II random mutagenesis kit (Agilent Technologies) under conditions resulting in a mutation frequency of up to 16 mutations per 1000 base pairs. After mutagenesis, a mixture of mutated genes was electroporated into LMG194 host cells containing the pWA23h plasmid. Typical mutant libraries consisted of more than 10^8^ independent clones. Bacterial cells were incubated overnight at 37 °C in LB medium supplemented with ampicillin and kanamycin.

To start protein expression 0.02% rhamnose and 0.004% arabinose were added. Cells were grown for 5 h at 37 °C, and then at 22 °C for 20 h. Before sorting, bacterial cells were washed with phosphate-buffered saline (PBS) and diluted with PBS to an optical density of 0.03 at 600 nm. Flow cytometry screening was performed on BD Influx cell sorter (BD Biosciences). Six hundred and forty nanometer laser for excitation and a 670/30 nm emission filter were used for selection of positive clones. Collected cells were rescued in SOC medium for 1 h at 37 °C, and then plated on LB/ampicillin/kanamycin Petri dishes supplemented with 0.004% arabinose and 0.02% rhamnose. Leica M205 FA fluorescence stereomicroscope equipped with a filter set ET CY5.5 (650/45 nm excitation and 710/50 nm emission filters) and a CCD camera (Tucsen) was used for screening of brightest clones. About 30 mutants selected in bacteria were then tested in HeLa cells, transfected with plasmids obtained after cloning of FP genes into pcDNA3.1(+) plasmid (Invitrogen/Thermo Fisher Scientific). A mixture of several selected mutants was then used as a template for the next round of mutagenesis.

### Protein expression and characterization

LMG194 bacterial cells, containing pWA23h plasmid encoding HO and pBAD/His-B plasmid encoding miRFP670nano with polyhistidine tag on the N termini were grown in LB medium supplemented with ampicillin and kanamycin. To induce HO expression 0.02% rhamnose was added. After incubation for 3 h at 37 °C, the expression of miRFP670nano was induced by 0.002% arabinose. Cells were cultured for 3 h at 37 °C and then at 22 °C for 20 h. For protein purification Ni-NTA agarose (Qiagen) was used. Protein was eluted with PBS containing 100 mM EDTA. For samples desalting PD-10 columns (GE Healthcare) were used.

Size-exclusion liquid chromatography of purified miRFP670nano was performed using HiLoad 16/600 Superdex 200 column (GE Healthcare) at a flow rate of 1 ml min^−1^. The column was equilibrated with 10 mM HEPES buffer, pH 7.4, containing 150 mM NaCl, 10% glycerol, 50 μM EDTA, 1 mM dithiothreitol, 0.2 mM phenylmethylsulfonyl fluoride, 0.01% EP-40, and 0.2 mM benzodiazepin. The column was calibrated with Bio-Rad gel filtration standards.

The fluorescence spectra were recorded with Cary Eclipse Fluorescence Spectrophotometer (Agilent Technologies), absorbance measurements were performed with Hitachi U-2000 spectrophotometer. The extinction coefficient of miRFP670nano was determined as a ratio between the absorbance value of the peak at Q-band and the value of the peak at Soret band, given a Soret band extinction coefficient of 39,900 M^−1^ cm^−1^^16^. The fluorescence quantum yield of miRFP670nano was determined using a Nile blue dye as a standard. pH stability was studied using a series of Hydrion buffers (Micro Essential Laboratory).

Maturation rate of FPs was compared in LMG194 bacterial cells expressing miRFP670nano or miRFP670, respectively. Cells were cultured in LB medium supplemented with ampicillin, kanamycin, and 0.2% rhamnose for 2 h at 37 °C. Then, protein expression was induced by 0.002% arabinose, and cells were incubated for 1 h at 37 °C. Next, cells were washed and resuspended in LB medium supplemented with ampicillin, kanamycin, and 0.2% rhamnose, but without arabinose. Cells were cultured at 37 °C for 8 h. Fluorescence intensity of the cell suspension was measured every 1 h. To normalize number of cells, aliquots of the cell suspension were diluted to the same optical density.

### Protein crystallization and structure determination

For crystallization the protein was dialyzed against 20 mM Tris-HCl, 200 mM NaCl at pH 8.0 buffer and concentrated to 28.4 mg ml^−1^. Crystallization conditions were found with Mosquito crystallization robot (TTP LabTech) using Hampton Research crystallization and additive screens. Crystals suitable for X-ray data collection were obtained by hanging drop method from 0.1 M sodium acetate at pH 4.0, 10 mM EDTA, 10% v/v isopropanol, and 22% w/v polyethylene glycol 6000. In large-scale crystallization experiment, 2 μl of the protein solution was mixed with 2 μl of the reservoir solution and incubated over 500 ml of the same reservoir solution at 20 °C for 2 weeks.

X-ray data were collected on SER-CAT 22-ID beamline (Advanced Photon Source, Argonne National Laboratory, Argonne, IL, USA) using standard equipment of the station. To minimize radiation damage the crystals were flash frozen in a 100 K nitrogen stream and a helical data collection technique was used for all X-ray data acquisitions. Diffraction images were processed with the HKL2000^41^. The statistics are given in Supplementary Table 2.

The structure of miRFP670nano was solved by the molecular replacement method with MOLREP^42^ using the structure of AnPixJ(PDB ID: 3W2Z^24^) in its red-absorbing state as a search model. To increase the contrast of rotation function the search model was truncated to the residues 36–183. To remove model bias, the chains were rebuilt with the PHENIX.AUTOBUILD crystallographic molecular model building suite^43,44^. Real space model correction was performed with COOT^45^, structure refinement was done with REFMAC^46^, and structure validation was carried out with COOT and PROCHECK^47^. The refinement statistics is given in Supplementary Table 3.

###  Design of mammalian plasmids

To construct mammalian expression plasmids, the respective genes of miRFP670nano or mutants were inserted in a pcDNA3.1 plasmid (Invitrogen/Thermo Fisher Scientific) by *Kpn*I/*Eco*RI sites.

For protein tagging and labeling of intracellular structures study, miRFP670nano was amplified, digested with restriction enzymes, and then swapped with miRFP703 either as C- (for α-tubulin and clathrin) or N-terminal fusions (for keratin, α-actinin, LifeAct, EB3, myosin, vimentin, clathrin, LAMP1, and H2B)^3^.

To engineer caspase-3 activity NIR-reporter, fusion of miRFPP670nano and miRFP720, containing 11 amino acid linker with the caspase-3 recognition site (GGDEVDGPVAT), was designed. For this, a miRFP670nano gene was PCR amplified using primers containing the linker sequence, *Nhe*I and *Age*I sites and inserted into pcDNA3.1 plasmid (Invitrogen/Thermo Fisher Scientific), then miRFP720 gene was inserted by *Age*I and *Not*I sites.

To create a JNK and PKA activity NIR-biosensor plasmids, we used a pJNKAREV-NES (3555NES) and pAKAR3EV-NES (3536NES) plasmids^29^ kindly provided by K. Aoki. A YPet gene was replaced with miRFP670nano gene by *Eco*RI/*Xho*I sites. An ECFP gene was replased with miRFP720 gene by *Not*I/*Xba*I sites. Then, fragments encoding NIR sensors were cut out with *Eco*RI and *Sal*I restriction endonucleases and inserted into pcDNA3.1 plasmid (Invitrogen/Thermo Fisher Scientific). Fragment encoding p38m-KTR was cut out from pLentiPGK Puro DEST p38KTRClover (a gift from Markus Covert (Addgene plasmid # 59152)) with *Eco*RI and *Age*I restriction endonucleases and inserted into pEGFP-N1 (Clontech). Venus-PA-PKI was a gift from Klaus Hahn (Addgene plasmid # 65456). OptoJNKi was cut out from OptopKCAG-mCherry-OptoJNKi (a gift from Michael Courtney (Addgene plasmid # 89738)) with *Eco*RI and *Bam*HI restriction endonucleases and inserted into pEGFP-C1 (Clontech).

### Mammalian cells and transfection

HeLa (CCL-2), U87 (HTB-14), U-2 OS (HTB-96), and NIH3T3 (CRL-1658) cells were obtained from the ATCC, PC6-3 cells were a kind gift of Dan Lindholm (University of Helsinki). Cells were grown in a Dulbecco's modified Eagle's medium (DMEM) medium supplemented with 10% fetal bovine serum, 0.5% penicillin–streptomycin, and 2 mM glutamine (Life Technologies/Invitrogen). For microscopy, cells were cultured in 35 mm glass-bottom Petri dishes (Greiner Bio-One International).

Plasmid transfections were performed using polyethylenimine^48^. Stably expressing cells were selected with 1 mg ml^−1^ G418 antibiotic. Sorting of positive cells was performed using a BD Influx cell sorter (BD Biosciences) equipped with 640 nm laser for excitation and a 670/30 nm emission filter.

### Cell fixation

HeLa cells transfected with miRFP670nano, miRFP670, and miRFP703 were dissociated from culture dishes with 0.25% trypsin (Gibco/Thermo Fisher Scientific), washed and re-suspended in PBS. For fixation, 10^6^ cells were incubated on ice with 1 ml of 4% paraformaldehyde solution for 10, 30, or 60 min and then washed. Fluorescence was measured using Cary Eclipse Fluorescence Spectrophotometer (Agilent Technologies).

### Neuronal culture and transfection

Primary rat cortical neurons were prepared in Neuronal Cell Culture Unit, University of Helsinki. All animal work was performed in accordance with the ethical guidelines of the European convention and regulations of an Ethics committee for animal research of the University of Helsinki. Cells were plated at a density of 600,000–700,000 per glass bottom 35 mm dishes coated with poly-l-Lysine (0.01 mg ml^−1^) (Merck) in a neurobasal medium (Gibco) supplemented with B27 (Life Technologies/Invitrogen), l-glutamine (Invitrogen), and penicillin-streptomycin (Lonza). Cultured neurons were transfected at 2–3 days in vitro (DIV) with a pcDNA3.1 plasmid (Invitrogen/Thermo Fisher Scientific), encoding miRFP670nano using Effectene Transfection Reagent (Qiagen). Neurons were imaged 48 h after transfection.

### Widefield fluorescence microscopy

Live cells were imaged with an Olympus IX81 inverted epifluorescence microscope 48 h after the transfection. The microscope was equipped with a 200 W metal halide arc lamp (Lumen220PRO, Prior), a 60 × 1.35 numerical aperture (NA) oil objective lens (UPlanSApo, Olympus) and an opiMOS sCMOS camera (QImaging). During imaging HeLa cells were incubated in a cell imaging medium (Life Technologies-Invitrogen) and kept at 37 °C. The microscope was operated with a SlideBook v.6.0.8 software (Intelligent Imaging Innovations). To separately image miRFP670nano and miRFP720 in one cell (two NIR color imaging), the two filter sets (605/30 nm exciter and 667/30 nm emitter, and 685/20 nm exciter and 725/40 nm emitter) (Chroma) were used.

Photobleaching measurements of cytoplasmically expressed NIR FPs in live HeLa cells were performed with the 60 × 1.35 NA oil immersion objective lens (UPlanSApo, Olympus) and a 650/13 nm (exciter) and 684/24 nm (emitter) filter set (Semrock).

To obtain FRET images a 605/30 excitation filter and two emission filters (667/30 nm for miRFP670nano and 725/40 nm for miRFP720) were used. Emission ratios were obtained by calculating background-subtracted FRET intensities divided by background-subtracted miRFP670nano intensities for JNK and PKA NIR biosensors. For caspase-3 reporter FRET to donor intensities ratio was calculated. FRET measurements were quantified using ImageJ (NIH). Intensity-modulated display mode was generated with a full-spectrum lookup table. Time-course ratio measurements were normalized to baseline prestimulation values. HeLa cells expressing JNK and PKA NIR biosensors were starved for 6 h with DMEM medium (Gibco/Thermo Fisher Scientific) before imaging.

To photoactivate PA-PKI and OptoJNKi, the transfected cells were continuously illuminated using 460/20 nm custom‐assembled LED array (LED Engin) at the light power density of 0.5 mW cm^−2^.

### Flow cytometry

The samples were analyzed using a BD Accuri C6 flow cytometer. Prior to acquisition, cell pellets were washed with PBS and diluted in cold PBS to a density of 500,000 cells ml^−1^. At least 50,000 cells per sample were recorded. miRFP670nano, miRFP670, and miRFP703 were excited with a 640 nm laser and their fluorescence was detected with a 675/25 or 670 nm LP emission filters. EGFP was excited with a 488 nm laser, and its fluorescence was detected with a 510/15 nm emission filter. Mean NIR fluorescent intensity of the double-positive cell population was normalized to mean green fluorescence intensity of the co-expressed EGFP to account for transfection efficiency. The data were analyzed using a FlowJo v.7.6.2 software.

### Imaging in mice

The Swiss Webster 2- to 3-month-old female mice (National Cancer Institute, NIH) with body weights of 22–25 g were used. To compare brightness of miRFP670nano with miRFP670 as well as to show possibility of two-color imaging HeLa cells were injected subcutaneously in the interscapular area of FVB mice. For better imaging, the fur on the bellies of the mice was removed using a depilatory cream. HeLa cells were co-transfected with the pcDNA-miRFP670nano or pcDNA-miRFP670 and pRluc8 plasmids in a 10:1 ratio for comparison study. HeLa cells were transfected with the pcDNA-miRFP670nano or pmiRFP720 and pRluc8 plasmids for two-color study. Various number of HeLa cells in 100 μl of RPMI-1640 media supplemented with 2 mM l-glutamine were injected subcutaneously 72 h after the transfection. For fluorescence and bioluminescence detection, 1 h after the HeLa cells injection the animals were imaged using an IVIS Spectrum instrument (Perkin Elmer/Caliper Life Sciences). Fluorescence was detected with 640/20 nm excitation and 680/30 nm emission filters for miRFP670nano or 675/20 nm excitation and 720/30 nm emission filters for miRFP720. Bioluminescence was detected with an open emission filter. Throughout the imaging, animals were maintained under anesthesia with 1.5% vaporized isofluorane. Prior to imaging, 80 μg of Inject-A-Lume coelenterazine substrate for Rluc8 (NanoLight Technology) was intravenously injected through a retro-orbital vein. Data were analyzed using Living Image 3.0 software (Perkin Elmer/Caliper Life Sciences).

All animal experiments were performed in an AAALAC-approved facility using protocols approved by the Albert Einstein College of Medicine Animal Usage Committee. Forty-five mice were used in this study.

### Reproducibility

The experiments were not randomized. The investigators were not blinded to allocation during the experiments and outcome assessment. No sample-size estimation was performed to ensure adequate power to detect a pre-specified effect size.

## Supplementary Information

Supplementary Information

Reporting Summary

Flow Cytometry Reporting Summary

## Data Availability

The main data supporting the findings of this study are available within the Article and its Supplementary materials. The additional data are available from the corresponding author on reasonable request. miRFP670nano nucleotide sequence in GenBank is MK176509. miRFP670nano crystal structure in Protein Data Bank is 6MGH.
