# Supplementary material for: Smallest near-infrared fluorescent protein evolved from cyanobacteriochrome as versatile tag for spectral multiplexing
Source: Nat Commun. 2019 Jan 17;10:279. doi: 10.1038/s41467-018-08050-8 (PMC6336887; doi:10.1038/s41467-018-08050-8)
Supplement: Supplementary file 1 — Supplementary Information [file 41467_2018_8050_MOESM1_ESM.pdf]

Smallest near-infrared fluorescent protein evolved from cyanobacteriochrome as versatile tag for spectral multiplexing

Olena S. Oliinyk, Anton A. Shemetov, Sergei Pletnev, Daria M. Shcherbakova, and Vladislav V. Verkhusha

Supplementary Information

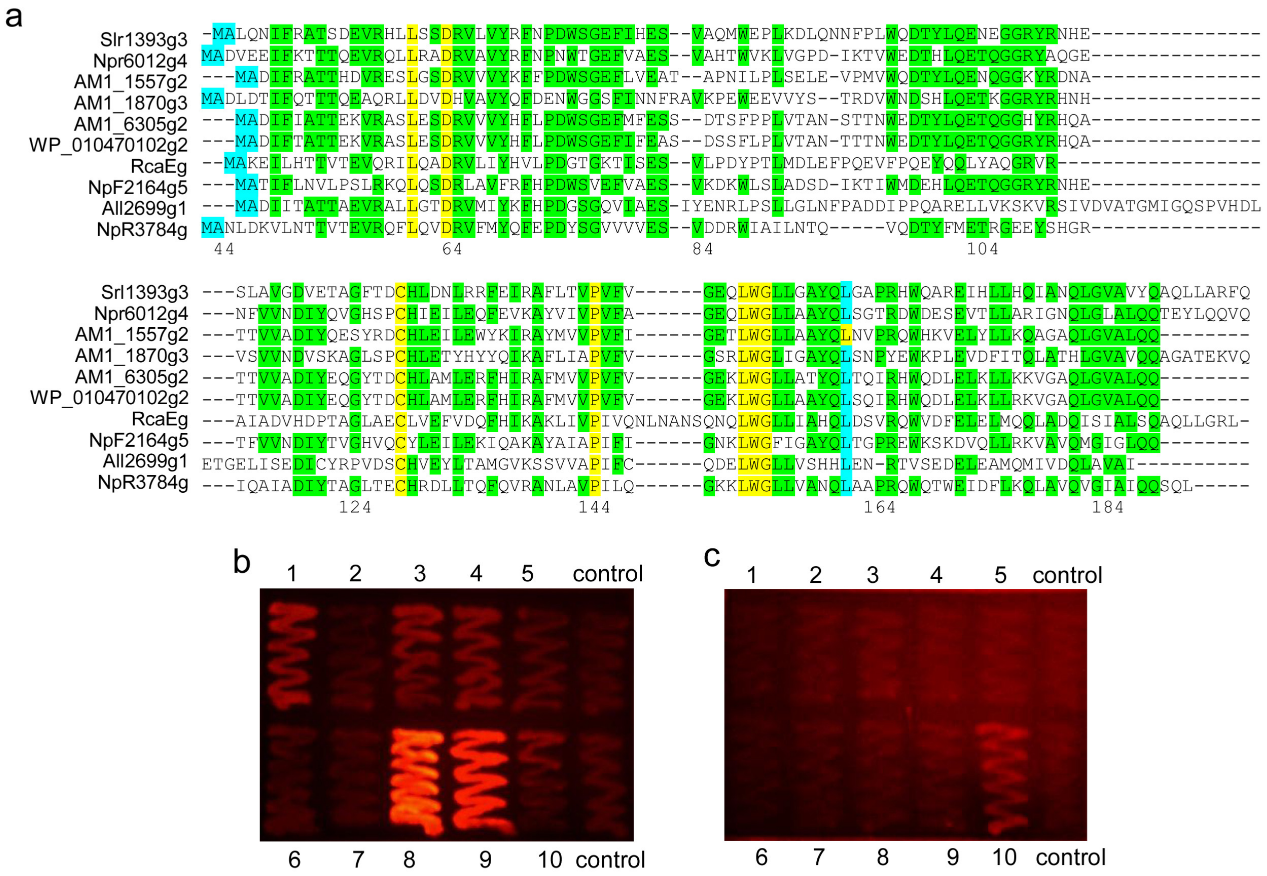

Supplementary Figure 1. Evaluation of CBCR GAF domains as templates for NIR FP. (a) Alignment of the amino acid sequences of evaluated CBCR GAF domains. Identical amino acid residues are highlighted in yellow, conserved amino acid residues are highlighted in green. Met and Ala added to N-terminus and introduced Leu are highlighted in blue. Numbering follows that for NpR3784 sequence. **(b)** CBCR GAF domains expressed in PCB-producing *E.coli*. **(c)** CBCR GAF domains expressed in BV-producing *E.coli*. 1 – slr1393g3 (a.a. 441-596); 2 – Npr6012g4 (a.a. 600-755); 3 – AM1_1557g2 (a.a. 220-364); 4 – AM1_1870g3 (a.a. 513-668); 5 – AM1_6305g2 (a.a. 240-384); 6 – WP_010470102g2 (a.a. 254-398); 7 – RcaEg (a.a. 115-271); 8 – NpF2164g5 (a.a. 873-1017); 9 – all2699g1 (a.a. 34-195); 10 – NpR3784g (a.a. 44-189); control – bacteria transformed with empty plasmid.

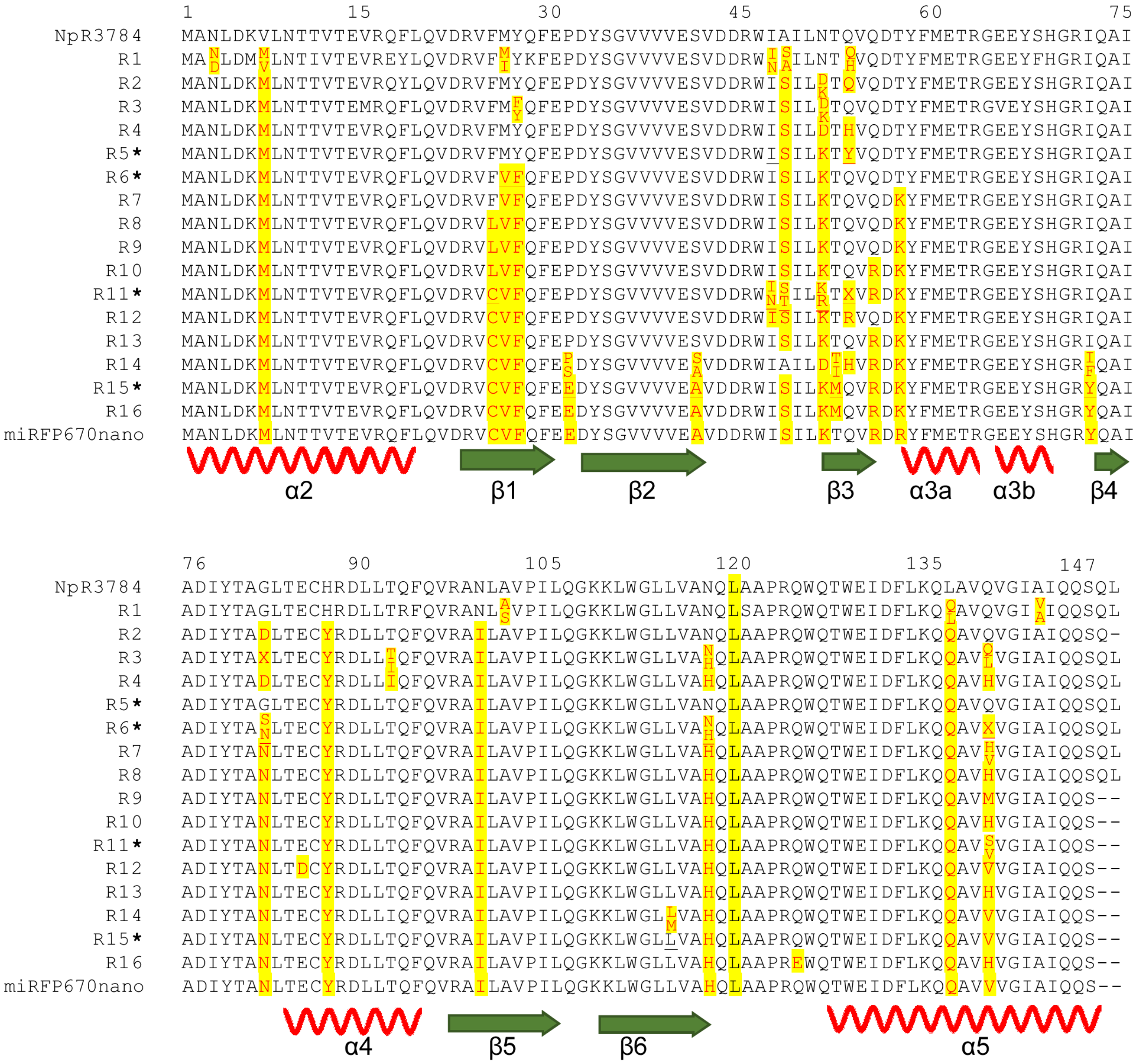

Supplementary Figure 2. Alignment of the amino acid sequences of clones selected on each round of molecular evolution. Saturated mutagenesis rounds indicated with asterisk and corresponding mutated position are underlined. Structural elements are based on the determined crystal structure of miRFP670nano (see text).

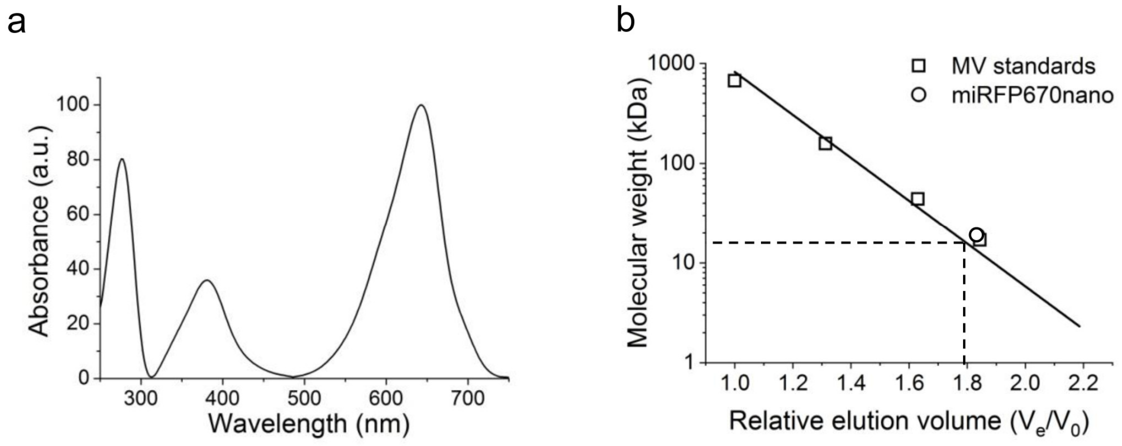

Supplementary Figure 3. Biochemical and photochemical properties of miRFP670nano. (a) Absorbance spectrum of miRFP670nano (apoprotein $A^{0.1\%}{}_{280}$ value = 1.79 AU). **(b)** Size exclusion chromatography calibration plot. V_e, elution volume; V_o, void volume of column.

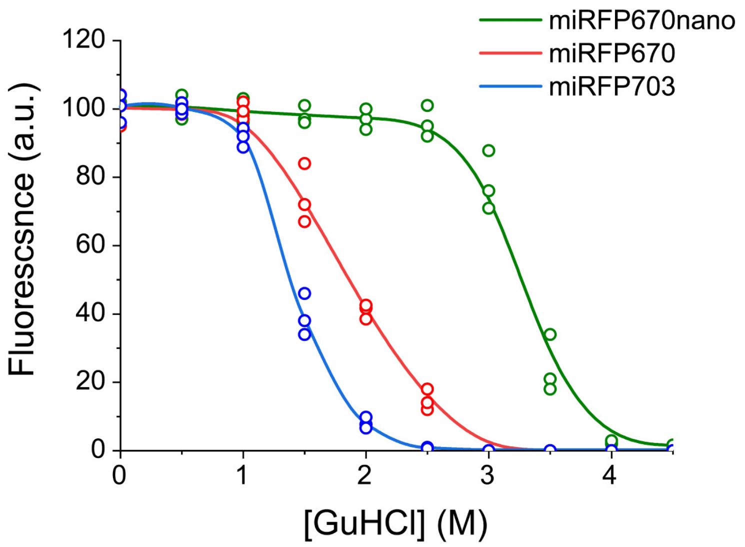

Supplementary Figure 4. Comparison of NIR FPs stabilities to denaturation condition. The fluorescence of NIR FPs after 24 h of incubation at different denaturant guanidine hydrochloride (GuHCl) concentrations. The data were normalized to the fluorescence of NIR FPs in buffered solution (*n*= 3; transfection experiments). Calculated concentrations of guanidine hydrochloride, in which 50% fluorescence is retained, are 3.3 M for miRFP670nano, 1.85 M for miRFP670 and 1.4 M for miRFP703.

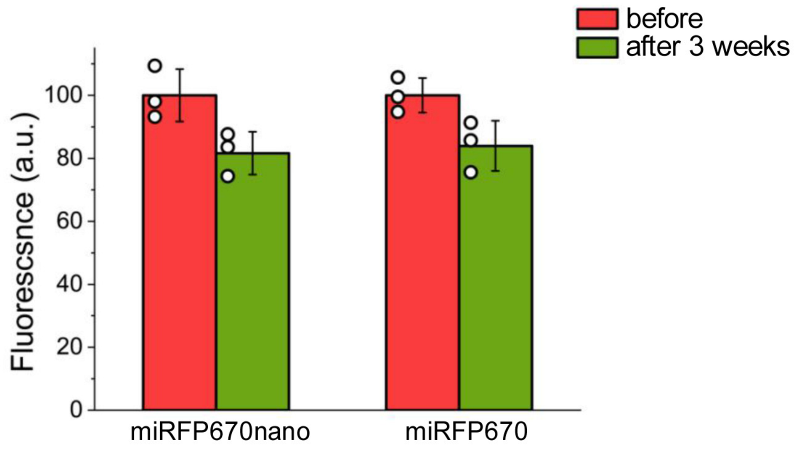

Supplementary Figure 5. Cytotoxicity assay. Mean fluorescence intensities of live HeLa cells stably expressing miRFP670nano and miRFP670 were analyzed by flow cytometry on day 14 (red) and day 35 (green) after transfection. Error bars, s.d (n=3 independent experiments).

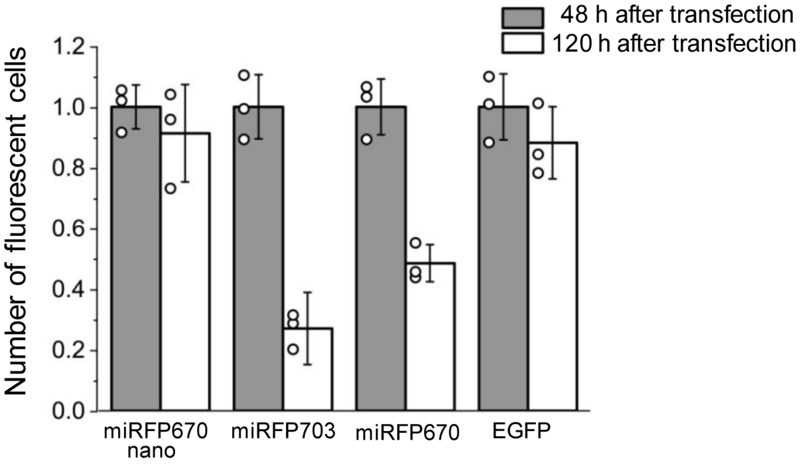

Supplementary Figure 6. Stability of miRFP670nano in transiently transfected HeLa cells. The number of FP expressing (fluorescent) HeLa cells transiently transfected with miRFP670nano, miRFP703, miRFP670 and EGFP was calculated 48 h and 120 h after transfection. The values were normalized to the percentage observed 48 h after transfection. Error bars, s.d. (*n*=3 transfection experiments).

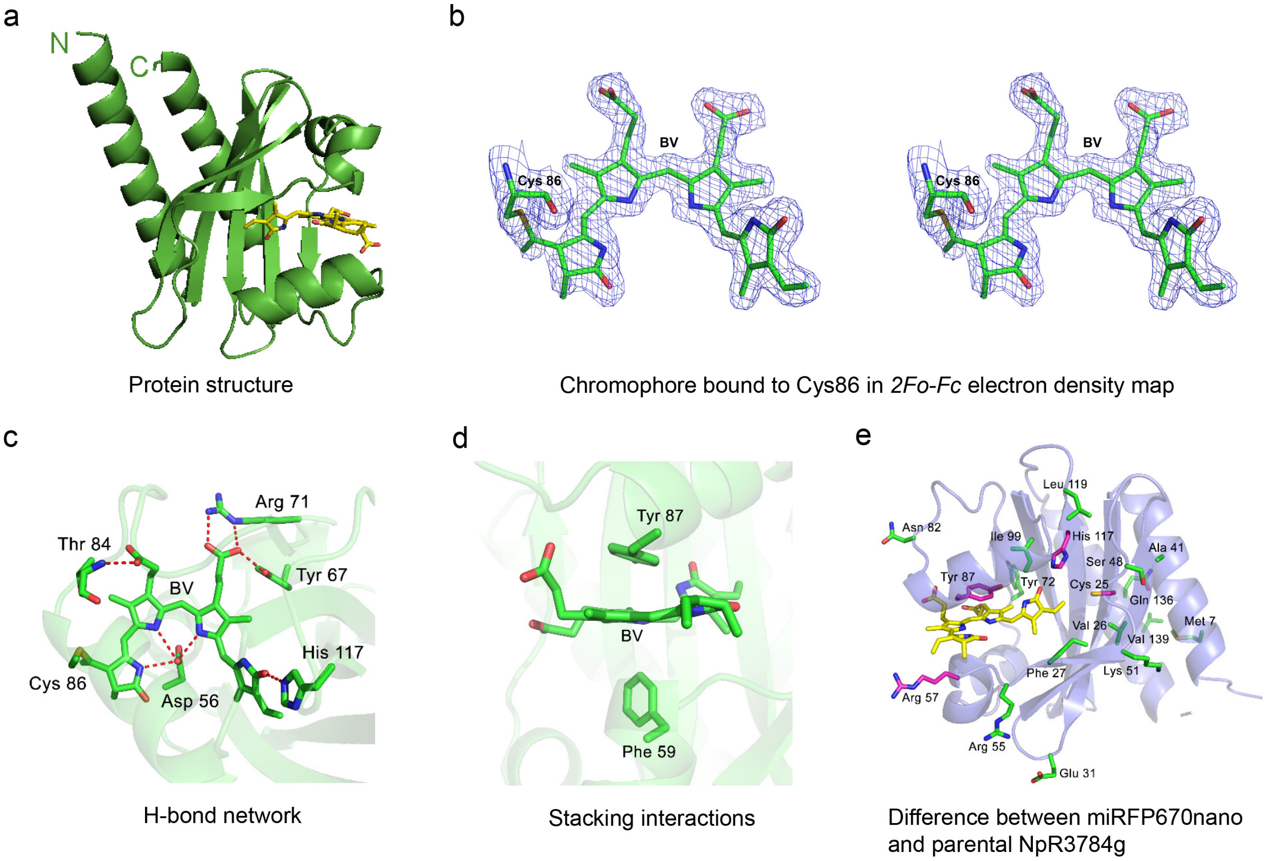

Supplementary Figure 7. miRFP670nano protein structure and its chromophore environment. (**a**) Overall structure of miRFP670nano. (**b**) The chromophore bound to Cys86 in 2*Fo-Fc* electron density map countered at 1.0σ. (**c**) Hydrogen bond network around the chromophore. (**d**) Stacking interactions between the chromophore and surrounding residues. BV adduct forms one parallel and one T-shaped stacking interaction with Y87 and F59, respectively. (**e**) Amino acid difference between miRFP670nano and parental CBCR NpR3784g. Residues that are different in miRFP670nano and NpR3784g are shown as sticks (both green and magenta). Residues that had the most impact on miRFP670nano spectral properties are shown in magenta.

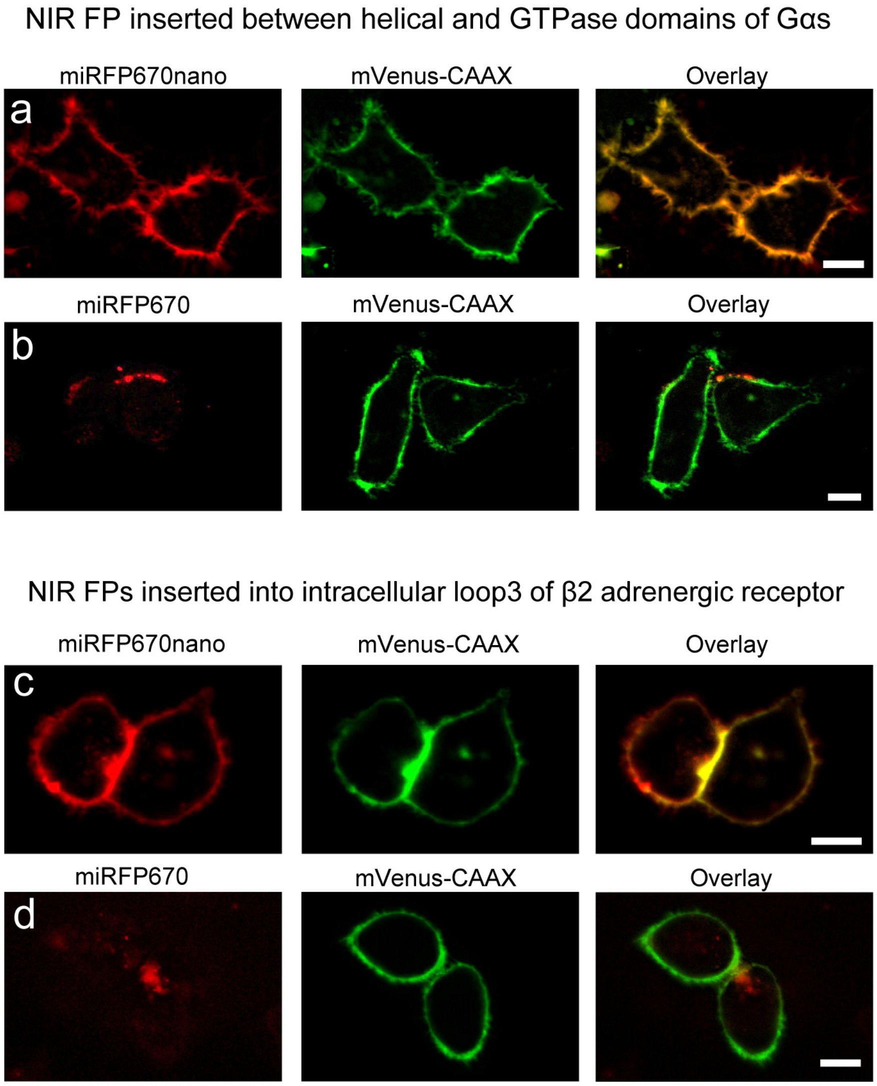

Supplementary Figure 8. miRFP670nano as internally inserted fluorescent tag. **(a)** miRFP670nano and **(b)** miRFP670 inserted between the helical and GTPase domains of the G protein α subunit. **(c)** miRFP670nano and **(d)** miRFP670 inserted into the intracellular loop 3 of the β2 adrenergic receptor. mVenus with membrane targeting CAAX motif used for membrane visualization. Scale bars, 10 μm.

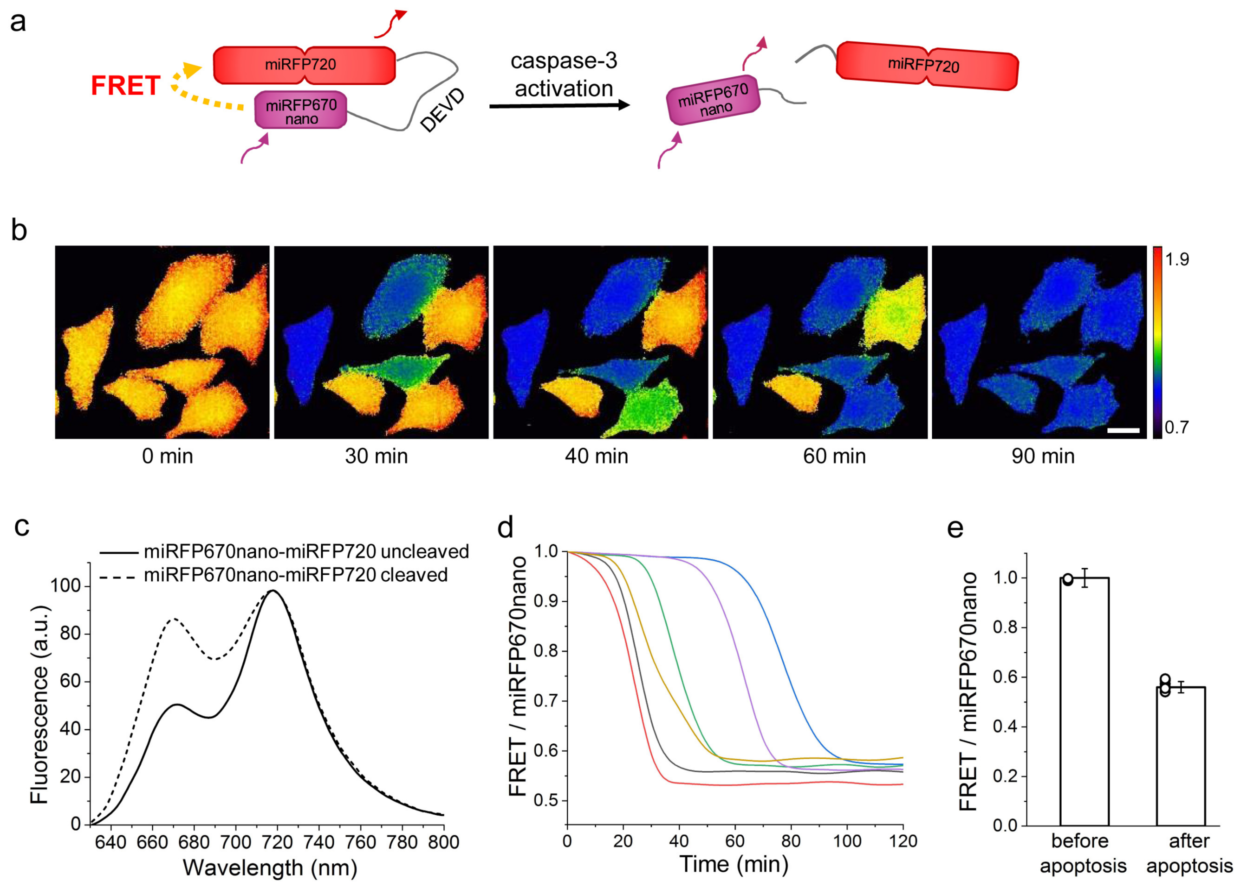

Supplementary Figure 9. miRFP670nano-DEVD-miRFP720 FRET-based reporter for caspase-3 activity (a) Schematic representation of the caspase-3 activity reporter consisting of miRFP670nano (FRET donor), 11 a.a. linker with DEVD caspase-3 cleavage site, and miRFP720 (FRET acceptor). **(b)** Time-lapse FRET/miRFP670nano ratio images of HeLa cell expressing miRFP670nano-DEVD-miRFP720 reporter upon apoptosis induced with 10 μM staurosporine, visualized using pseudocolor. **(c)** Emission spectra of miRFP670nano-DEVD-miRFP720 reporter before and after cleavage. **(d)** FRET/miRFP670nano ratio time courses of individual cells undergoing apoptosis. **(e)** FRET/miRFP670nano ratio before and after staurosporine-induced apoptosis. Error bars, s.d. (*n*=3; independent experiments). Scale bar, 10 μm.

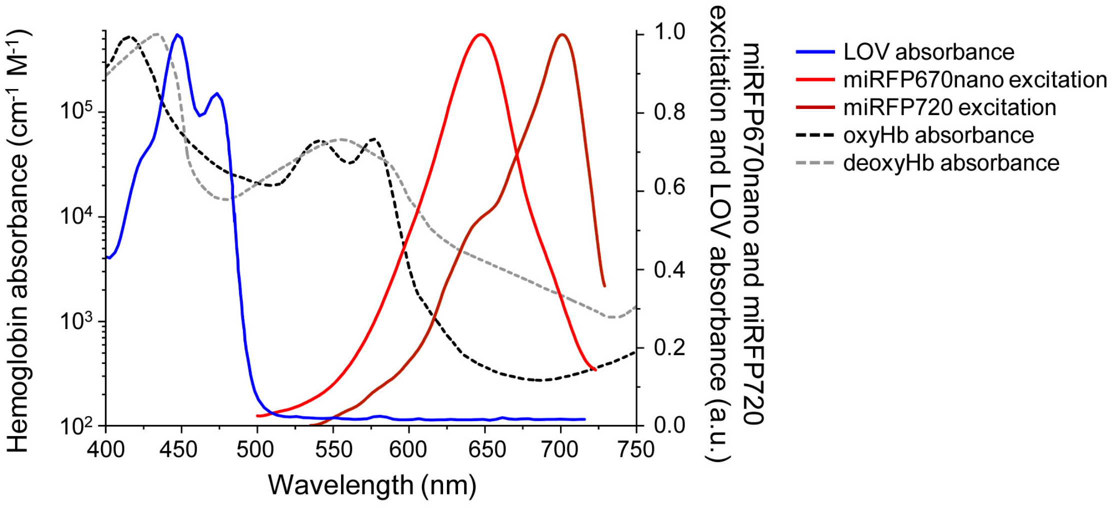

Supplementary Figure 10. Non-overlapping spectra of LOV2 domain and miRFP670nano-miRFP720 FRET pair. Spectra of miRFP670nano and miRFP720 are located in the NIR tissue transparency window (650-900 nm) where the extinction coefficient of both oxyhemoglobin (oxyHb) and deoxyhemoglobin (deoxyHb) are 1-2 orders of magnitude lower than in the blue-green spectral range.

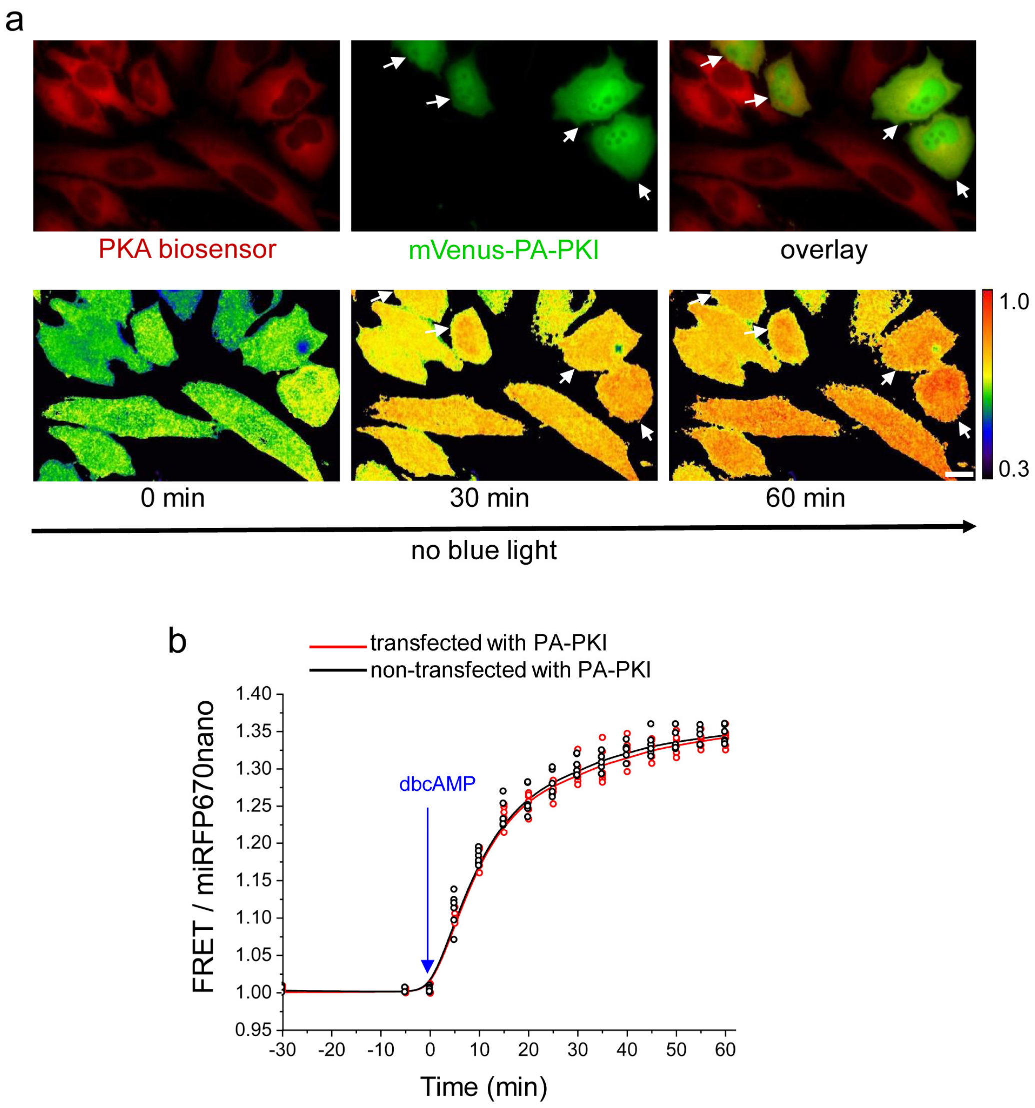

Supplementary Figure 11. Control experiments to evaluate spectral compatibility of NIR PKA biosensor with optogenetic PKA inhibitor. (a) HeLa cells stably expressing NIR PKA biosensor co-transfected with photoactivatable PKA inhibitor, PA-PKI, tagged with mVenus (top row). Upon stimulation with 1 mM dbcAMP without illumination with blue light, the changes in the FRET/miRFP670nano ratio are shown in pseudocolor (bottom row). **(b)** FRET/miRFP670nano ratio time courses of HeLa cells expressing NIR PKA biosensor only (black) or NIR PKA biosensor with PA-PKI (red) upon stimulation with 1 mM dbcAMP without illumination with blue light (*n*=3 independent experiments). White arrows indicate cells expressing the optogenetic inhibitor. Scale bar, 10 μm.

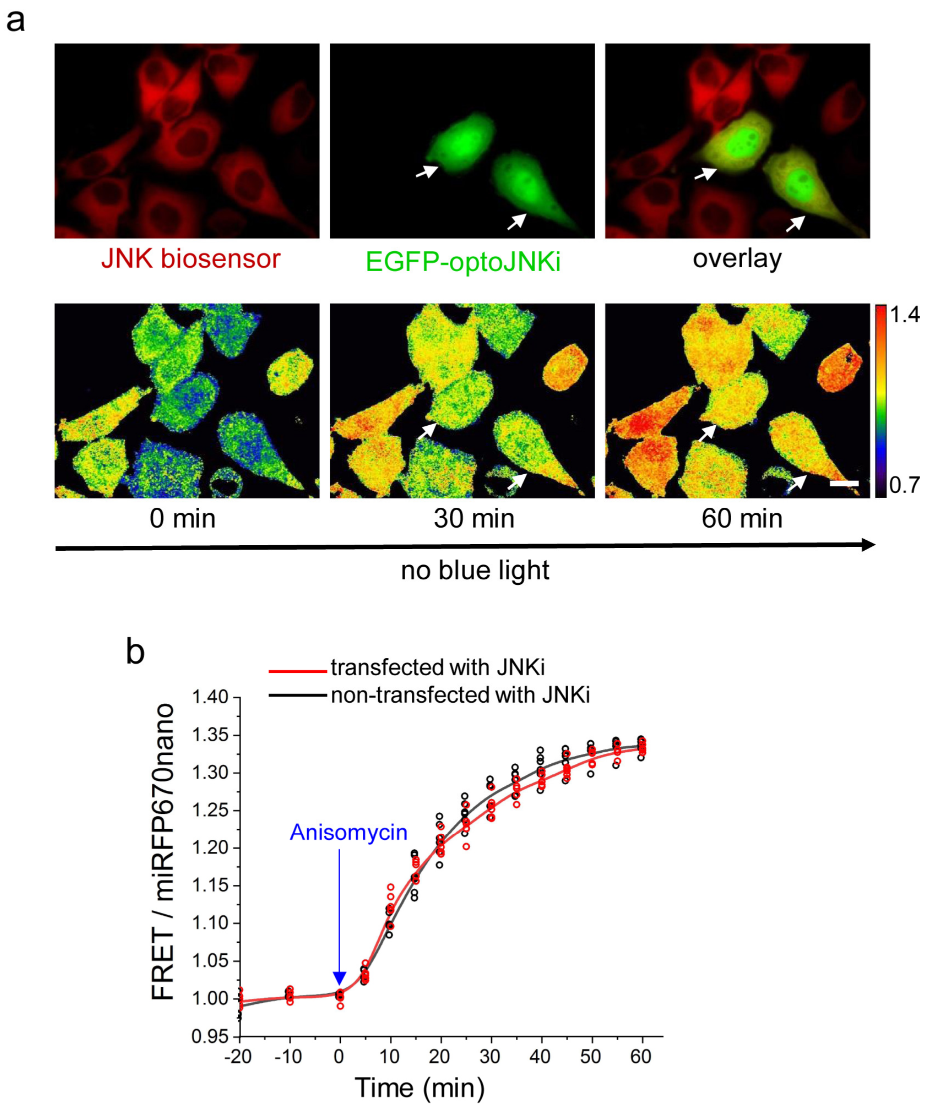

Supplementary Figure 12. Control experiments to evaluate spectral compatibility of NIR JNK biosensor with optogenetic JNK inhibitor. (a) HeLa cells stably expressing NIR JNK biosensor co-transfected with optogenetic JNK inhibitor, optoJNKi, tagged with EGFP (top row). Upon stimulation with 1 µg/ml anisomycin without illumination with blue light, the changes in the FRET/miRFP670nano ratio are shown in pseudocolor (bottom row). **(b)** FRET/miRFP670nano ratio time courses of HeLa cells expressing NIR JNK biosensor only (black) or NIR JNK biosensor with optoJNKi (red) upon stimulation with 1 µg/ml anisomycin without illumination with blue light (n=3 independent experiments). White arrows indicate cells expressing the optogenetic inhibitor. Scale bar, 10 µm.

Supplementary Table 1. List of primers.

Primer	Primer sequence (5'-3')
NpR3784-KpnI For	ATCTGCAGCTGGTACCATGG
NpR3784-EcoRI	GCTTCGAATTCAGCTAGCTCTG
pBAD For	ATGCCATAGCATTTTTATCC
pBAD Rev	GATTTAATCTGTATCAGG
CMV For	AAATGGGCGGTAGGCGTGTAC
pcDNA Rev	TAGAAGGCACAGTCGAGGC
miRFP670nano-KpnI For	AAAGGTACCATGGCAAACCTGGACAAG
miRFP670nano-EcoRI Rev	AAAGAATTCAGCTAGCTCTGCTGGATGGCGATGC
For G93/X	GACATCTACACCGCANNSCTGACAGAGTGCTACC
Rev G93/X	GGTAGCACTCTGTCAGSNNTGCGGTGTAGATGTC
For Leu25/X	GCAGGTGGACAGAGTGNNSGTGTTCCAGTTTGAGC
Rev Leu25/X	GCTCAAACTGGAACACSNNCACTCTGTCCACCTGC
For M37/X Y38/X	GGTGGACAGAGTGTTCNNSNNSCAGTTTGAGCCAGATTATAGC
Rev M37/X Y38/X	GCTATAATCTGGCTCAAACTGSNNSNNGAACACTCTGTCCACC
For N128/X	GGCCTGCTGGTGGCANNSCAGCTGGCAGCCCCTAG
Rev N128/X	CTAGGGGCTGCCAGCTGSNNTGCCACCAGCAGGCC
For Q147/X	CAAGCAGCAGGCCGTGNNSGTGGGCATCGCCATCC
Rev Q147/X	GGATGGCGATGCCCACSNNCACGGCCTGCTGCTTG
For P31/X	GCGTGTTCCAGTTTGAGNNSGATTATAGCGGAGTGGTGG
Rev P31/X	CCACCACTCCGCTATAATCSNNCTCAAACTGGAACACGC
For S41/X	GGAGTGGTGGTGGTGGAGNNSGTGGACGATAGGTGGATCTCC
Rev S41/X	GGAGATCCACCTATCGTCCACSNNCTCCACCACCACCACTCCGC
For T52/X	GGATCTCCATCCTGAAGNNSCAGGTGCGGGATAAATAC
Rev T52/X	GTATTTATCCCGCACCTGSNNCTTCAGGATGGAGATCC
For I72/X	GGAGTATTCTCACGGCCGCNNSCAGGCCATCGCCGACATCTAC

Rev I72/X	GTAGATGTCGGCGATGGCCTGSNNGCGGCCGTGAGAATACTCC
For L114/X	GCAAGAAGCTGTGGGGCCTGNNSGTGGCACACCAGCTGGCG
Rev L114/X	CGCCAGCTGGTGTGCCACSNNCAGGCCCCACAGCTTCTTGC
Cys25 For	GCAGGTGGACAGAGTGTGCGTGTTCCAGTTTGAGCC
Cys25Rev	GGCTCAAACTGGAACACGCACACTCTGTCCACCTGC
3X For	GGMDCHMSATCCTGRVSACCVVSNNSNNSNNSAAATACTTYATGGAGACAAGGG
3X Rev	CCCTTGTCTCCATRAAGTATTTSNNSNNSNNSBBGGTSBYCAGGATSKDGHKCC
4X For	NNSNNSATCCTGNNSACCNNSGTGCAGGATACATACTTCATGG
4X Rev	CCACCTATCGTCCACGGAC
For Y98/X	CAAACCTGACAGAGTGCNNSCGGGATCTGCTGACACAG
Rev Y98/X	CTGTGTCAGCAGATCCCGSNNGCACTCTGTCAGGTTTG
miRFP670nano-N-terminal fusions For	AAACCGGTAATGGCAAACCTGGACAAG
miRFP670nano-N-terminal fusions Rev	ATAGGTACCGTACTCGTCCTGGTCTTC
miRFP670nano-C-terminal fusions For	AAGCTAGCGGTACCATGGCAAACC
miRFP670nano-C-terminal fusions Rev	TTAGATCTTCCTCCTGATCCACCACCTCCAGAACCACCTCCTCCTGATCCACCACCTCCGCTCTGCTGGATGGCGATGCCC
miRFP670nano-actin For	TATAGATCTATGGATGATGATATCGCCGCG
miRFP670nano-actin Rev	ATATCTAGACTAGAAGCATTTGCGGTGG
miRFP670nano-linker-BamHI For	AAGGATCCGGTGGGGGAGGCAGCATGGCAAACCTGGACAAGATGC
miRFP670nano-linker-AgeI Rev	TTACCGGTGCTCCCGCCACCTCCGCTCTGCTGGATGGCGATGC
Gas-1 For	AAAGCTAGCATGGGCTGCCTCGGCAACAG
Gas-1 Rev	TTTGGATCCCTCTCCGTTAAACCC
Gas-2 For	TTACCGGTGATGGTGAGAAGGCCACCAAAGTGC
Gas-2 Rev	TTTGCGGCCGCTTAGAGCAGCTCGTATTGGCG

beta2AR-1 For	AAGCTAGCATGGGGCAACCCGGGAACGGCAGCG
beta2AR-2 Rev	TTGGATCCCTCCACCTGGCTAAGGTTCTGG
beta2AR-2 For	AAACCGGTCAGGATGGGCGGACGGGGC
beta2AR-2 Rev	TTGCGGCCGCTTACAGCAGTGAGTCATTTGTAC
miRFP720-Age For	TATACCGGTCGCCACCATGGCGG
miRFP720-Age Rev	ATACCGGTCCATCAACTTCATCTCCTCCCTCTTCCATCACGCCG
miRFP720-Nhe For	AAAGCTAGCATGGCGGAAGGATCCGTCGCC
BglI Rev	TCGAGATCTTCCTCCTGATCCACCACCTCC
C-miRFP720 For	CATGGACTAGCGCTACCGGTCGCCACCATGGCGGAAGGATCCGTCGCC
C-miRFP720 Rev	AAAGGGCCCCTCACTCTTCCATCACGCCG
N-miRFP670nano AKAR For	AAAGAATTCATGGCAAACCTGGACAAG
N-miRFP670nano AKAR Rev	TTTCTCGAGGCTCTGCTGGATGGCG
C-miRFP720 AKAR For	AAAGCGGCCGCATGGCGGAAGGATCCGTCGCC
C-miRFP720 AKAR Rev	TTTTCTAGACTCACTCTTCCATCACGCCG
miRFP720-XbaI Rev	AATCTAGACTCTTCCATCACGCCGATC
p38 For	TTGAATTCTGCAGATATCAACAAGT
p38 Rev	AAACCGGTGTGCTGGACTGGAGGGTCAGG

Supplementary Table 2. Data collection statistics.

**Protein**	**miRFP670nano**
Space group	*C*2
Unit cell parameters (Å, º)	a = 187.6
	b = 72.1
	c = 136.7
	β = 130.0
Temperature (K)	100
Wavelength (Å)	1.00
Resolution (Å)	30.0 – 1.95
Total reflections	376,413
Unique reflections	100,484
Completeness (%)	98.5 (97.6)
I/σ<I>	13.4 (2.0)
R-merge	0.097 (0.56)
Multiplicity	3.7 (3.3)

Data in parentheses are given for the outermost resolution shell 2.02 – 1.95 Å

Supplementary Table 3. Refinement statistics.

Protein	miRFP670nano
No. of protein atoms	10,041
No. of solvent atoms	584
Resolution range (Å)	30.0 – 1.95
R-work	0.183
R-free	0.239
R.m.s.d. bond lengths (Å)	0.019
R.m.s.d. angles (º)	2.42
R.m.s.d. chirality (º)	0.15
R.m.s.d. planarity (º)	0.011
R.m.s.d. dihedral (º)	21.6
Mean B factors ($Å^2$)	
Protein atoms	
overall	26.8
main chain	24.0
side chain	29.3
chromophore	29.8
Water	33.0
Ramachandran statistics (%) (for non-Gly/Pro residues)	
most favorable	90.5
additional allowed	9.3
generously allowed	0.1
disallowed	0.1
