# Supplementary material for: Smallest near-infrared fluorescent protein evolved from cyanobacteriochrome as versatile tag for spectral multiplexing
Source: Nat Commun. 2019 Jan 17;10:279. doi: 10.1038/s41467-018-08050-8 (PMC6336887; doi:10.1038/s41467-018-08050-8)
Supplement: Supplementary file 3 — Flow Cytometry Reporting Summary [file 41467_2018_8050_MOESM3_ESM.pdf]

nature research

Corresponding author(s): Vladislav Verkhusha

☐ Initial submission ☐ Revised version ☒ Final submission

Flow Cytometry Reporting Summary

Form fields will expand as needed. Please do not leave fields blank.

Data presentation

For all flow cytometry data, confirm that:

☒ 1. The axis labels state the marker and fluorochrome used (e.g. CD4-FITC).

☒ 2. The axis scales are clearly visible. Include numbers along axes only for bottom left plot of group (a 'group' is an analysis of identical markers).

☐ 3. All plots are contour plots with outliers or pseudocolor plots.

☒ 4. A numerical value for number of cells or percentage (with statistics) is provided.

Methodological details

5. Describe the sample preparation.	Prior to acquisition, cell pellets were washed with PBS and diluted in cold PBS to density 500,000 cells per ml . At least 50,000 cells per sample were recorded.
6. Identify the instrument used for data collection.	BD Accuri C6 flow cytometer
7. Describe the software used to collect and analyze the flow cytometry data.	CFlow Plus, FlowJo v.7.6.2
8. Describe the abundance of the relevant cell populations within post-sort fractions.	Not applicable
9. Describe the gating strategy used.	Initial gates - FSC-A/SSC-A to discriminate cells from debris; then cells were gated in FSC-W/FSC-A to discriminate single cells; then cells were gated in SSC-W/SSC-A to discriminate live cells. Resulted population were analyzed on SSC-A/FL1 and SSC-A/FL4 plots to find cells expressing EGFP and NIR FPs. Mock transected cells were used for selecting negative population.

Tick this box to confirm that a figure exemplifying the gating strategy is provided in the Supplementary Information. ☐
